# Pharmacological Efficacy of Intravenous Magnesium in Attenuating Remifentanil-Induced Postoperative Hyperalgesia: A Systematic Review and Meta-Analysis of Randomized Controlled Trials

**DOI:** 10.3390/ph18040518

**Published:** 2025-04-01

**Authors:** En-Bo Wu, Kuen-Lin Wu, Wei-Ti Hsu, Wei-Chin Yuan, Kuen-Bao Chen

**Affiliations:** 1 Department of Anesthesiology, China Medical University Hospital, China Medical University, Taichung City 404, Taiwan; enbofive@gmail.com (E.-B.W.); 006339@tool.caaumed.org.tw (W.-T.H.); evelynyuan48@gmail.com (W.-C.Y.); 2 Division of Colorectal Surgery, Department of Surgery, Kaohsiung Chang-Gung Memorial Hospital, Chang-Gung University College of Medicine, Kaohsiung City 833, Taiwan; focus913@gmail.com; 3 Department of Anesthesiology, School of Medicine, China Medical University, Taichung City 404, Taiwan

**Keywords:** magnesium, remifentanil, opioid-induced hyperalgesia, analgesic requirements, postoperative pain, meta-analysis

## Abstract

**Background/Objectives**: Remifentanil-based anesthesia is linked to opioid-induced hyperalgesia (OIH), increasing postoperative pain and analgesic requirements. Magnesium, an N-methyl-D-aspartate (NMDA) receptor antagonist, might alleviate OIH. We aimed to assess whether intravenous magnesium reduces postoperative pain, analgesic requirements, and hyperalgesia in adults receiving remifentanil-based anesthesia. **Methods**: We searched PubMed, Embase, the Cochrane Library, and Web of Science (1 December 2024) for randomized controlled trials (RCTs) comparing intravenous magnesium vs. placebo. Risk of bias was evaluated with the Cochrane RoB 2 tool, and random-effects meta-analyses were conducted. GRADE was used to assess evidence quality. Primary outcomes were postoperative analgesic requirements and pain scores; secondary outcomes included intraoperative remifentanil consumption, shivering, postoperative nausea/vomiting (PONV), extubation time, hypotension, and bradycardia. PROSPERO registration: CRD42024609911. **Results**: Twenty-two RCTs (*n* = 1362) met eligibility. Magnesium significantly decreased 24 h analgesic requirements (standardized mean difference [SMD] −1.51; 95% confidence interval [CI] −2.15 to −0.87; *p* < 0.0001) and pain scores (SMD −0.61; 95% CI −0.90 to −0.32; *p* < 0.0001), with benefits persisting up to 48 h. It also reduced intraoperative remifentanil use (SMD −0.52), shivering (odds ratio [OR] 0.25), and PONV (OR 0.66), without prolonging extubation or increasing hypotension/bradycardia risk. High heterogeneity, potential publication bias, and moderate-to-very-low evidence certainty warrant caution. **Conclusions**: Intravenous magnesium appears beneficial in remifentanil-based anesthesia, but further large-scale, methodologically robust trials are needed to confirm optimal and clarify safety profiles across diverse surgical populations.

## 1. Introduction

Remifentanil, a μ-opioid receptor agonist with an ultra-short duration of action, is frequently selected for clinical anesthesia due to its rapid onset, adjustable dosing, and favorable pharmacokinetic profile. These properties help sustain stable intraoperative conditions and enable expedient postoperative recovery. Nonetheless, accumulated clinical evidence suggests that administering high doses of remifentanil during surgery may intensify postoperative pain sensitivity and raise analgesic requirements [1,2]. Such an outcome, commonly attributed to opioid-induced hyperalgesia (OIH), accentuates the importance of novel interventions aimed at minimizing postoperative discomfort in remifentanil-based anesthetic protocols.

**Figure 1:**
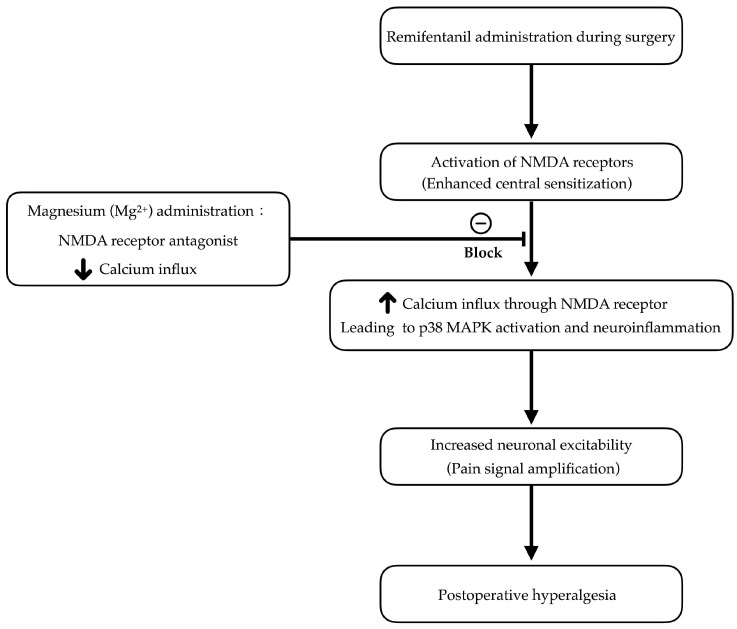
Proposed mechanism by which magnesium mitigates remifentanil-induced postoperative hyperalgesia. NMDA, N-methyl-D-aspartate; MAPK, mitogen-activated protein kinase.

Central sensitization, predominantly involving N-methyl-D-aspartate (NMDA) receptor pathways, has been identified as a pivotal mechanism underlying opioid-related hyperalgesia [2]. Excessive NMDA receptor activity not only heightens nociceptive transmission but may also engage the p38 mitogen-activated protein kinase (p38 MAPK) cascade, thus exacerbating postoperative pain [3]. In this context, magnesium, a physiological NMDA receptor antagonist, has attracted considerable attention for its potential to moderate hyperalgesia processes [4]. By modulating NMDA receptor excitation, magnesium could suppress central sensitization, reduce postoperative pain intensity, and possibly curtail perioperative opioid requirements [5,6] (Figure 1). Emerging data further indicate that magnesium’s analgesic and anti-inflammatory attributes may provide broader perioperative advantages [6]. Even so, some investigations have found its benefits to be inconsistent. For instance, in a systematic review of multiple randomized controlled trials (RCTs), Lysakowski et al. [7] observed that magnesium did not confer uniformly positive analgesic outcomes, suggesting a high degree of variability in patient populations and protocols. This lack of consensus leaves the precise function of magnesium in remifentanil-based anesthesia open to question.

Several meta-analyses have addressed the effects of magnesium on postoperative pain within remifentanil-based regimens, yet their conclusions diverge. Liu et al. (2012) reported that NMDA receptor antagonists (including magnesium sulphate) did not significantly diminish postoperative pain or analgesic usage, which could stem from heterogeneity in study designs and limited sample sizes [8]. Conversely, Wu et al. (2015) observed reduced pain scores and analgesic requirements, although their analysis predated 2015 publications and lacked comprehensive subgroup assessments [9]. More recently, Yue et al. (2022) conducted a meta-analysis confined to spinal surgery populations and reported that magnesium showed promising analgesic benefits in this context [10]. Nevertheless, focusing exclusively on spine procedures inevitably limits the applicability of their conclusions to broader surgical settings. These discrepancies, combined with variations in magnesium dosing regimens, patient populations, and outcome measures, maintain the uncertainty surrounding magnesium’s efficacy under remifentanil-based anesthesia.

We therefore conducted a comprehensive meta-analysis of contemporary RCTs to assess whether intravenous magnesium meaningfully reduces postoperative analgesic requirements and pain intensity in remifentanil-based anesthesia. In addition, we evaluated secondary outcomes, such as hemodynamic stability, incidence of postoperative nausea and vomiting (PONV), shivering, and extubation times. By exploring these endpoints across diverse patient cohorts and surgical contexts, our aim was to determine magnesium’s clinical efficacy, safety, and optimal dosing strategies to prevent or attenuate OIH.

## 2. Materials and Methods

To ensure comprehensive and transparent reporting, we conducted this systematic review and meta-analysis in accordance with the PRISMA (Preferred Reporting Items for Systematic Reviews and Meta-Analyses) guidelines [11]. Our methodological approach was further guided by the Cochrane Handbook for Systematic Reviews of Interventions (version 6.5; last updated August 2024) [12]. The study protocol was registered on PROSPERO (registration no. CRD42024609911) and remains publicly accessible at https://www.crd.york.ac.uk/prospero/, accessed on 25 March 2025. After the initial registration, we removed our plan to analyze Quality of Recovery (QoR) scores as these data were not systematically reported in the eligible trials. The amendment date, rationale, and specific revisions are detailed in the PROSPERO records.

### 2.1. Search Strategy

On 1 December 2024, two authors (E.-B.W. and K.-B.C.) conducted a comprehensive search of four electronic databases (PubMed, Embase, the Cochrane Library, and Web of Science) for English-language human studies examining magnesium’s role in remifentanil-induced hyperalgesia. The following Boolean logic was employed: PubMed: #1 = (“magnesium”[MeSH Terms] OR “magnesium”[All Fields] OR “magnesiums”[All Fields] OR “magnesiums”[All Fields]); #2 = (“remifentanil”[MeSH Terms] OR “remifentanil”[All Fields] OR “remifentanils”[All Fields]); #3 = (“hyperalgesia”[MeSH Terms] OR “hyperalgesia”[All Fields] OR “hyperalgesias”[All Fields]); #4 = #2 OR #3; #5 = #1 AND #4; #6 = filters: English, Humans. Embase: #1 = ‘magnesium’/exp OR magnesium; #2 = ‘remifentanil’/exp OR remifentanil; #3 = ‘hyperalgesia’/exp OR hyperalgesia; #4 = #2 OR #3; #5 = #1 AND #4; #6 = limits: English, Human. Cochrane Library: #1 = magnesium:ti,ab,kw; #2 = (remifentanil OR hyperalgesia):ti,ab,kw; #3 = #1 AND #2; #4 = filter: English. Web of Science: #1 = TS = (magnesium); #2 = TS = (remifentanil OR hyperalgesia); #3 = #1 AND #2; #4 = filter: English. All retrieved results were then exported to a reference manager and de-duplicated prior to further evaluation.

### 2.2. Selection Criteria

We included only RCTs that compared an intravenous magnesium infusion (intervention group) with saline placebo (control group) in patients undergoing remifentanil-based anesthesia. Studies were excluded if they employed any non-intravenous magnesium route (e.g., oral or local infiltration); did not use a remifentanil-based protocol; or were non-randomized, observational, animal-based, or published solely as conference abstracts, reviews, or meta-analyses. Two reviewers (E.-B.W. and K.-L.W.) independently examined all titles and abstracts; subsequently, full texts were reviewed for eligibility, and any discrepancies were settled in discussion with a third reviewer (W.-T.H.).

### 2.3. Data Extraction

Two authors (E.-B.W. and K.-L.W.) independently performed data extraction in accordance with a standardized protocol. For each eligible trial, the following data were recorded: first author’s name, year of publication, participant groups and sample sizes, participant age and sex distribution, American Society of Anesthesiologists (ASA) Physical Status classification and surgical type, details of the magnesium intervention, initial remifentanil infusion rates, premedication and anesthetic maintenance agents, postoperative analgesics and administration routes, any available trial registration identifier, and the relevant primary and secondary outcomes (postoperative analgesic requirements, postoperative pain scores, intraoperative hypotension and bradycardia incidence, extubation time, number of patients requiring rescue analgesia, incidence of shivering, incidence of PONV, patient satisfaction scores, and intraoperative remifentanil consumption). In cases where studies presented outcomes solely in graphical representations, numerical values were extracted using WebPlotDigitizer version 4.7.0 (Automeris, Redwood City, CA, USA), thereby facilitating consistent comparisons across datasets. Any discrepancies were resolved in consultation with a third reviewer (W.-T.H.).

### 2.4. Risk of Bias Assessment and Evidence Grading

We appraised the methodological quality of each included RCT using the Risk of Bias 2 (RoB 2) tool, as outlined in Chapter 8 of the Cochrane Handbook [12]. Two reviewers (E.-B.W. and K.-L.W.) independently assessed the randomization process, deviations from intended interventions, missing outcome data, outcome measurement, selective reporting, and overall risk of bias. Any disagreements were resolved through discussion with a third reviewer (W.-T.H.). Following these RoB 2 evaluations, studies were categorized as having “low risk”, “some concerns”, or “high risk” of bias. We then applied the Grading of Recommendations Assessment, Development and Evaluation (GRADE) framework [13,14] to determine the overall certainty of the evidence for each outcome, classifying it as high, moderate, low, or very low.

### 2.5. Outcome Measures and Data Standardization

Where studies presented outcomes as mean ± standard deviation (SD), these figures were directly extracted for the meta-analysis. If an RCT used median (interquartile range [IQR]), median (range), mean (95% confidence interval [CI]), or mean rank (generally adopted for skewed or nonparametric data), we converted them into mean ± SD using established algorithms [15,16,17]. All statistical procedures were independently reviewed by an experienced statistician (W.-C.Y.) to ensure methodological rigor and validity. In addition, trials that lacked SD values or provided only statements of statistical significance, without numerical data, were excluded to maintain consistency and support robust quantitative comparisons.

### 2.6. Data Synthesis and Analysis

All statistical evaluations were conducted using Review Manager (RevMan) version 5.4 (The Cochrane Collaboration, London, UK), MedCalc^®^ Statistical Software version 22.009 (MedCalc Software Ltd., Ostend, Belgium), and Comprehensive Meta-Analysis software version 3 (Biostat, Englewood, NJ, USA). For continuous variables (e.g., postoperative pain scores and extubation time), effect sizes were estimated as standardized mean differences (SMDs) with 95% confidence intervals (CIs). Dichotomous endpoints (e.g., incidence of PONV, shivering, and hypotension) were calculated as odds ratios (ORs) with 95% CIs. A random-effects model [18] was applied for the primary analyses. Heterogeneity was assessed via the Chi-squared test and the I^2^ statistic, with values below 50% considered low and those at or above 50% indicative of high heterogeneity.

Two pre-specified sensitivity analyses were undertaken to evaluate the robustness of the findings, as follows: (1) excluding trials deemed at high risk of bias (to reduce potential distortion of effect estimates due to methodological flaws), and (2) repeating all analyses under a fixed-effects model (to assess the stability of the pooled effects under different statistical assumptions). We did not apply additional corrections for multiple comparisons as the secondary analyses were exploratory. All statistical tests were two-sided, and *p* < 0.05 was considered significant.

### 2.7. Planned Subgroup Analyses

Subgroup analyses planned in advance were undertaken to explore potential sources of heterogeneity among the included trials and determine whether two principal factors [(1) surgical approach and (2) magnesium dosing strategy] might modify the effect of magnesium on key postoperative analgesic outcomes.

### 2.8. Assessment of Publication Bias

Publication bias was assessed only for outcomes supported by at least ten RCTs, given the limited interpretative value of funnel plots when fewer than ten studies are available [19]. For these outcomes, funnel plots were visually inspected for symmetry, and Egger’s test (*p* < 0.05) was used to detect any significant asymmetry [20]. Outcomes informed by fewer than ten RCTs were not evaluated for publication bias.

## 3. Results

**Figure 2:**
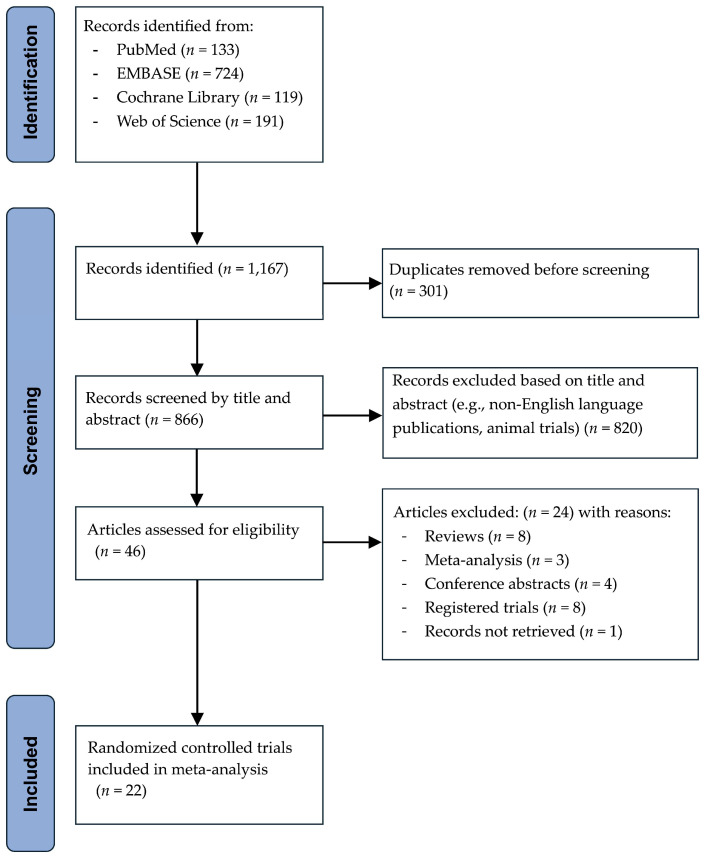
PRISMA flow diagram summarizing the literature search, screening, and final inclusion of 22 randomized controlled trials in the meta-analysis.

The initial database search identified 1167 records of which 301 were removed as duplicates before screening. An additional 820 citations were excluded upon title and abstract review because they were non-English articles, animal studies, or lacked relevance to the research question. Subsequently, 46 articles underwent full-text assessment, leading to the exclusion of 8 reviews, 3 meta-analyses, 4 conference abstracts, 8 registered trials without posted results, and 1 unobtainable document. Ultimately, 22 RCTs [4,21,22,23,24,25,26,27,28,29,30,31,32,33,34,35,36,37,38,39,40,41] fulfilled the eligibility criteria. No further relevant publications were identified through manual searches of reference lists. The overall identification and selection process adhered to the PRISMA guidelines (Figure 2).

### 3.1. Study Characteristics

The 22 included RCTs involved a total of 1362 participants who received remifentanil-based anesthesia, with 679 assigned to magnesium and 683 to saline (Table 1). These trials spanned publication years from 2001 through 2024 and were geographically dispersed, as follows: 1 was conducted in the United States [31]; Germany [21], Belgium [22], Austria [23], and Greece [32] each contributed 1 study; Turkey [24,26,27,29,37,40] contributed 6 studies; South Korea [4,25,28,33,35] 5; China [34,38,39,41] 4; and Iran [30,36] 2. All enrolled patients were categorized as ASA physical status I to III. Magnesium dosing strategies ranged from a single bolus to a bolus followed by continuous infusion. The surgical procedures studied were diverse, including pars plana vitrectomy, septorhinoplasty, thyroidectomy, laparoscopic cholecystectomy, robotic radical prostatectomy, rhinoplasty, major lumbar orthopedic surgery, cardiac surgery, major abdominal surgery, lumbar disc surgery, total abdominal hysterectomy, lower abdominal laparotomy, posterior spinal fusion for idiopathic scoliosis, lumbar laminectomy, spine surgery, radical mastectomy, orthognathic surgery, and lumbar spine surgery.

Although both the magnesium and placebo arms were documented in each trial, only these two groups were used in the statistical analyses. Additional information, such as the initial remifentanil infusion rate, premedications, anesthesia maintenance protocols, and postoperative analgesic regimens, was also extracted (Table 1). Ten of the RCTs reported prior trial registration; 4 were listed on ClinicalTrials.gov [28,31,32,33], 4 on the Chinese Clinical Trial Registry [34,38,39,41], 1 on the Korean Clinical Trial Registry [35], and 1 on the Iranian Registry of Clinical Trials [36].

### 3.2. Risk of Bias

**Figure 3:**
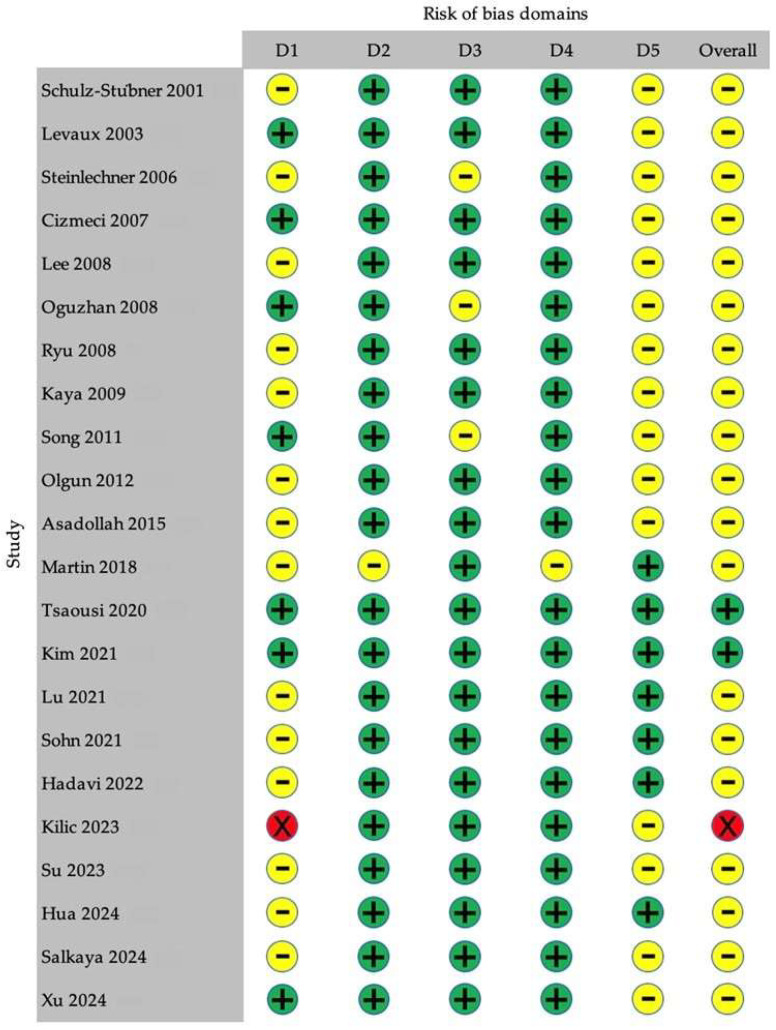
Traffic-light summary of the risk-of-bias assessment for the included trials carried out using the Cochrane Risk-of-Bias Tool for Randomized Trials (version 2; RoB 2). The color-coded circles indicate the level of concern; green denotes low risk, yellow signifies some concerns, and red represents high risk of bias. RoB, risk of bias. References [4,21,22,23,24,25,26,27,28,29,30,31,32,33,34,35,36,37,38,39,40,41].

Using the Cochrane RoB 2 tool, two studies were deemed at low risk of bias [32,33], most were evaluated as having “some concerns” [4,21,22,23,24,25,26,27,28,29,30,31,34,35,36,38,39,40,41], and one trial (Kilic et al., 2023) was judged to be at high risk of bias [37]. A graphical summary of these assessments is presented in Figure 3.

### 3.3. Primary Outcomes

The primary outcomes of interest in this meta-analysis were postoperative analgesic requirements and postoperative pain scores as these endpoints offer key insights into magnesium’s putative analgesic benefits when used alongside remifentanil-based anesthesia. The following sections present the pooled findings for both measures, highlighting temporal trends and summarizing the extent of heterogeneity observed across included trials.

#### 3.3.1. Postoperative Analgesic Requirements

This outcome encompassed all analgesics administered for postoperative pain control (type, dosage, and route). Measured doses were generally recorded in milligrams (mg) or micrograms (mcg), with mg·kg^−1^ used directly where reported. Analgesic agents included nalbuphine, metamizole, piritramide, remifentanil, morphine, meperidine, hydromorphone, fentanyl, and tramadol. Some RCTs quantified consumption via patient-controlled analgesia (PCA) volumes [4,25], PCA presses [41], or morphine milligram equivalents [32,33].

**Figure 4:**
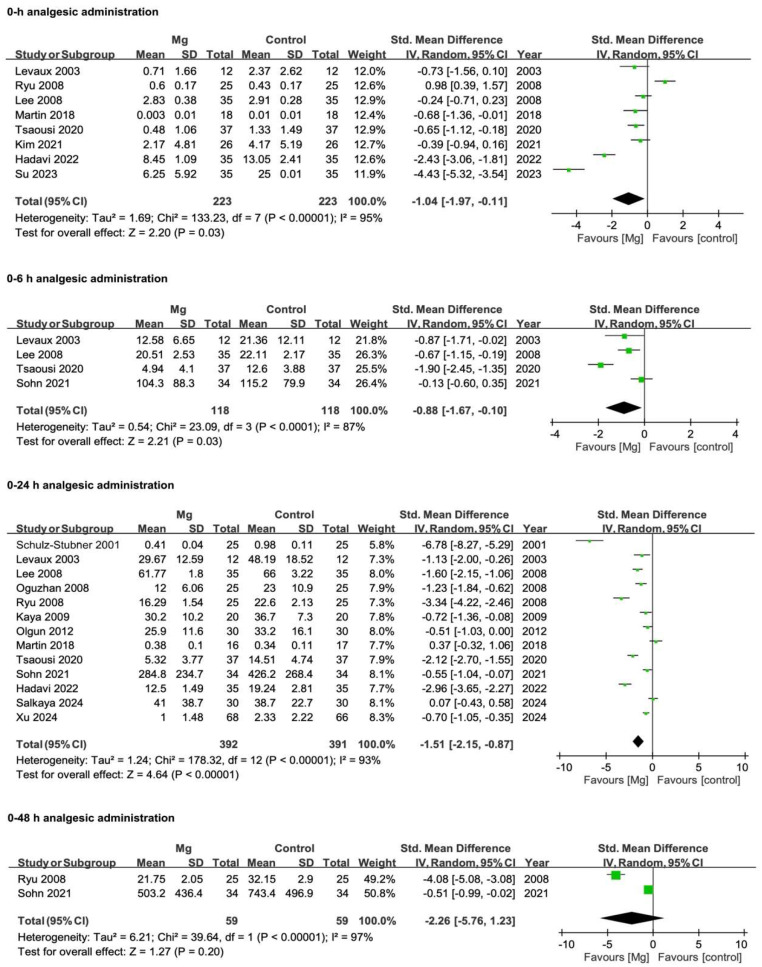
Forest plots illustrating the differences in postoperative analgesic administration between Mg and control at 0 h, 0–6 h, 0–24 h, and 0–48 h. Effect sizes are reported as SMDs with 95% CIs, derived from a random-effects model using the IV method. Negative values indicate reduced analgesic requirements with Mg, whereas positive values favor the control. Squares represent individual study estimates (weighted by sample size) and diamonds represent the pooled effect. Mg, magnesium; CI, confidence interval; IV, inverse variance; SD, standard deviation; SMD, standardized mean difference; Std., standard. References [4,21,22,25,26,27,29,31,32,33,35,36,38,40,41].

Across the included studies (Figure 4), magnesium consistently decreased analgesic requirements compared with controls at specific time points. At 0 h, the pooled SMD was −1.04 (95% CI −1.97 to −0.11; *p* = 0.03; I^2^ = 95%), suggesting a notable reduction in immediate analgesic requirements. Similar findings emerged for 0–6 h (SMD −0.88, 95% CI −1.67 to −0.10; *p* = 0.03; I^2^ = 87%) and 0–24 h (SMD −1.51, 95% CI −2.15 to −0.87; *p* < 0.0001; I^2^ = 93%). By contrast, at 0–48 h (SMD −2.26, 95% CI −5.76 to 1.23; *p* = 0.20; I^2^ = 97%), no significant difference was observed, although overall heterogeneity remained high. In general, these results indicate that magnesium may meaningfully reduce analgesic requirements over the first 24 h postoperatively, albeit with considerable variability across trials.

#### 3.3.2. Postoperative Pain Scores

Postoperative pain intensity was measured using instruments such as the Numeric Analogue Scale (NAS), Visual Analogue Scale (VAS), Verbal Rating Scale (VRS), Verbal Numeric Rating Scale (VNRS), or Numeric Rating Scale (NRS). Two RCTs demonstrated statistically significant differences favoring magnesium but did not provide extractable numerical data [21,22]; thus, they were included qualitatively but excluded from the meta-analysis of pain scores. In the remaining 20 RCTs, most studies employed a 0–10 scale, although a small number used a 0–100 VAS [4,23]. Where both rest- and effort-related scores were reported, only rest-state values were extracted [4]. Five reference time points (0, 6, 12, 24, and 48 h) were applied to ensure consistency.

**Figure 5:**
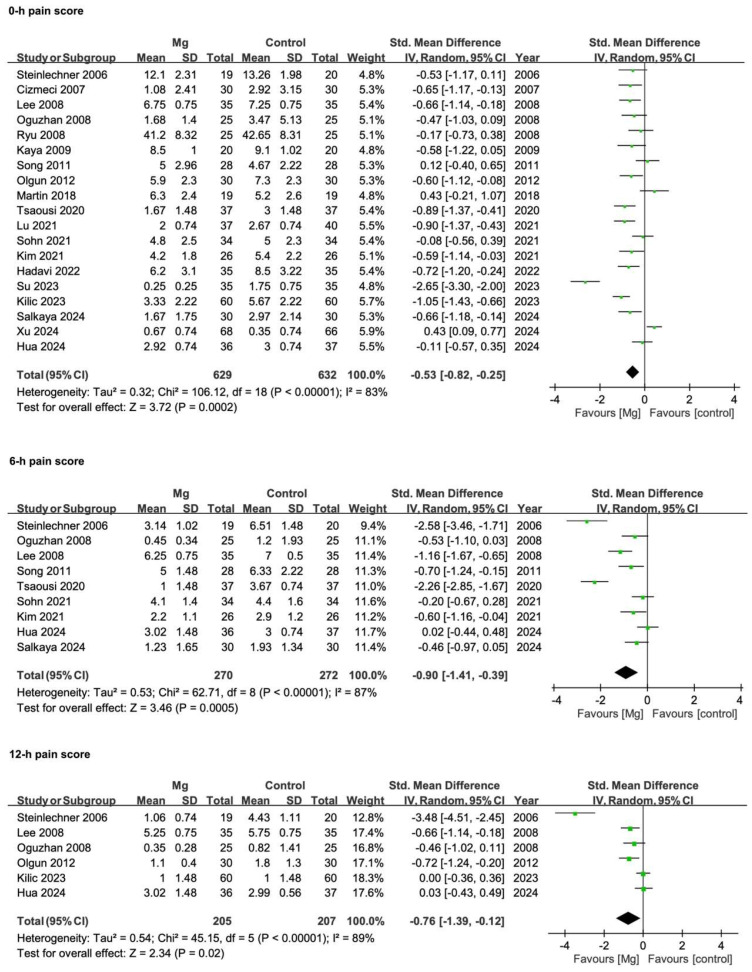

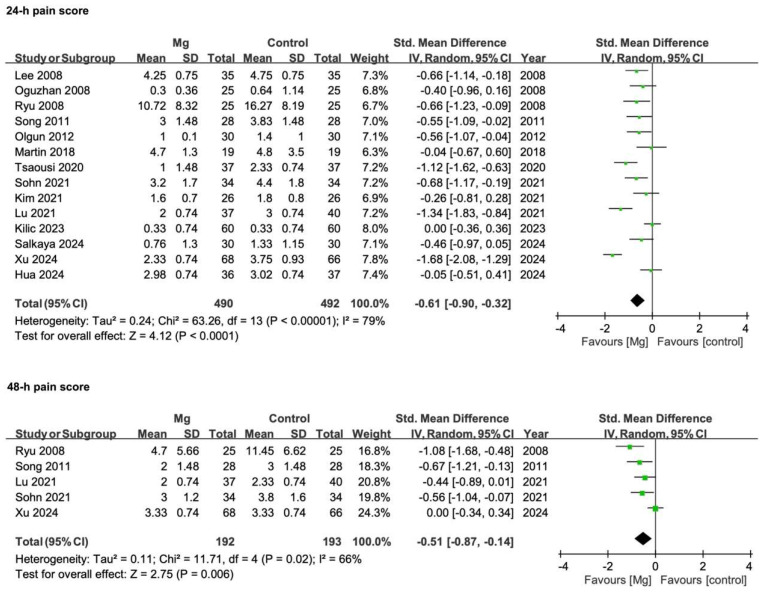
Forest plots illustrating the effect of Mg vs. control on postoperative pain scores at 0 h, 6 h, 12 h, 24 h, and 48 h. Each outcome is presented as a SMDs with 95% CIs, derived from a random-effects model using the IV method. Negative values indicate lower pain scores with Mg, whereas positive values favor the control group. Squares represent individual study estimates (weighted by sample size) and diamonds represent the pooled effect at each time point. CI, confidence interval; IV, inverse variance; Mg, magnesium; SD, standard deviation; SMD, standardized mean difference; Std., standard. References [4,23,24,25,26,27,28,29,31,32,33,34,35,36,37,38,39,40,41].

As shown in Figure 5, the pooled SMD at 0 h was −0.53 (95% CI −0.82 to −0.25; *p* = 0.0002; I^2^ = 83%), reflecting a moderate decrease in early postoperative pain. This benefit became more pronounced by 6 h (SMD −0.90, 95% CI −1.41 to −0.39; *p* = 0.0005; I^2^ = 87%), persisted at 12 h (SMD −0.76, 95% CI −1.39 to −0.12; *p* = 0.02; I^2^ = 89%) and 24 h (SMD −0.61, 95% CI −0.90 to −0.32; *p* < 0.0001; I^2^ = 79%), then slightly declined by 48 h (SMD −0.51, 95% CI −0.87 to −0.14; *p* = 0.006; I^2^ = 66%). Collectively, these findings indicate that magnesium confers a notable analgesic advantage, particularly during the initial recovery phase, although it continues to offer modest yet statistically significant pain relief up to 48 h.

### 3.4. Secondary Outcomes

**Figure 6:**
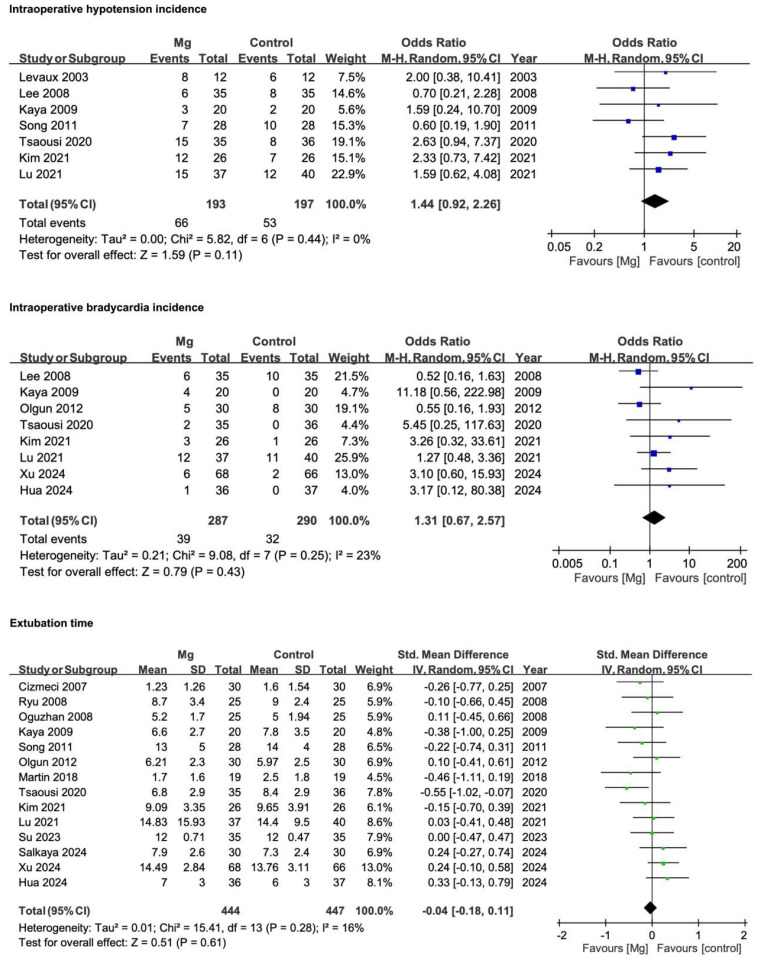

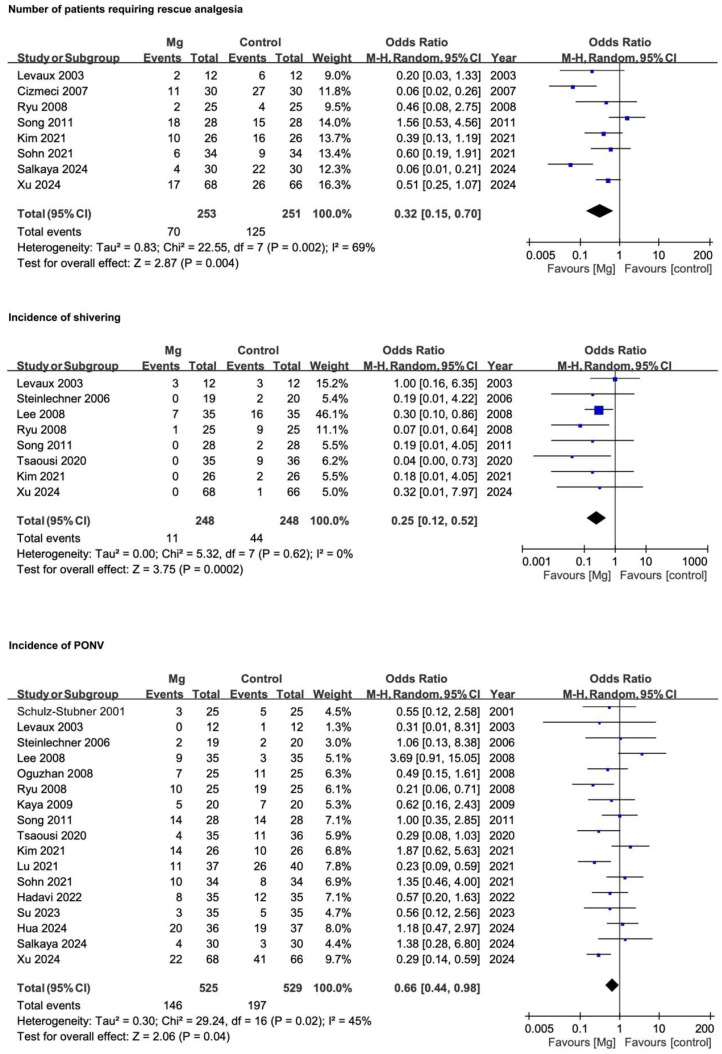

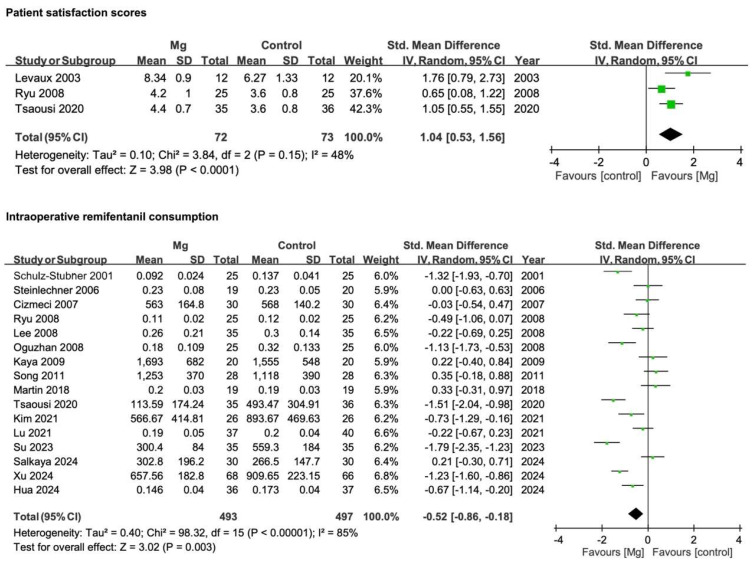
Forest plots comparing Mg with the control for intraoperative hypotension and bradycardia incidence, extubation time, number of patients requiring rescue analgesia, shivering, PONV, patient satisfaction, and intraoperative remifentanil consumption. Dichotomous outcomes are expressed as ORs with 95% CIs and continuous outcomes are presented as SMDs, all derived from a random-effects model. The M–H method was used for dichotomous data and the IV method was used for continuous data. Squares represent individual study estimates (weighted by sample size) and diamonds indicate the pooled effects. CI, confidence interval; IV, inverse variance; M–H, Mantel–Haenszel; Mg, magnesium; PONV, postoperative nausea and vomiting; SD, standard deviation; SMD, standardized mean difference; Std., standard. References [4,21,22,23,24,25,26,27,28,29,31,32,33,34,35,36,38,39,40,41].

In addition to the primary endpoints, several secondary measures were evaluated, including intraoperative hemodynamic parameters, extubation time, rescue-analgesia requirements, shivering, PONV, patient satisfaction, and intraoperative remifentanil consumption. Full definitions and methodological details of these outcomes are described below, followed by a summary of the pooled results (Figure 6).

#### 3.4.1. Intraoperative Hypotension Incidence

Intraoperative hypotension was defined according to each RCT’s specified threshold for blood pressure reduction or by the need for vasopressor administration (e.g., ephedrine or norepinephrine). Fifteen studies investigated hypotension but three provided only statistical conclusions without numerical data [21,26,41] and five documented zero events in both magnesium and control arms [4,23,24,39,40], leaving seven RCTs for meta-analysis [22,25,27,28,32,33,34]. Overall, the magnesium group did not show a significant increase in hypotension (OR 1.44, 95% CI 0.92–2.26; *p* = 0.11; I^2^ = 0%).

#### 3.4.2. Intraoperative Bradycardia Incidence

Bradycardia was defined by each study’s stated heart rate threshold (usually < 60 beats/min) or the need for atropine. Of 12 RCTs that examined intraoperative bradycardia [3,31,36,37,38], one provided no extractable data [21] and three reported zero bradycardic events in both groups [4,24,40]. Therefore, eight RCTs were included in the meta-analysis. Similar to hypotension, no significant difference emerged in bradycardia incidence (OR 1.31, 95% CI 0.67–2.57; *p* = 0.43; I^2^ = 23%).

#### 3.4.3. Extubation Time

Extubation time was recorded by 15 RCTs, defined as the interval from anesthesia cessation to endotracheal tube removal. One study reported implausibly high values, likely converted from seconds to minutes upon re-evaluation [33]. After excluding this anomaly, 14 RCTs remained. The pooled SMD was −0.04 (95% CI −0.18 to 0.11; *p* = 0.61; I^2^ = 16%), indicating no discernible difference between the magnesium and control groups.

#### 3.4.4. Number of Patients Requiring Rescue Analgesia

Eight RCTs reported the number of patients needing supplemental analgesics beyond the primary postoperative regimen, though timing of data collection varied [28,35,40]. The meta-analysis revealed a notably reduced odds ratio in the magnesium cohort (OR 0.32, 95% CI 0.15–0.70; *p* = 0.004; I^2^ = 69%), reflecting a lower likelihood of requiring additional analgesia.

#### 3.4.5. Incidence of Shivering

Postoperative shivering (or “chills”) was recorded in eight RCTs, which documented the number of patients experiencing this complication. The combined analysis demonstrated a clear advantage for magnesium (OR 0.25, 95% CI 0.12–0.52; *p* = 0.0002; I^2^ = 0%), suggesting a protective effect against remifentanil-related shivering.

#### 3.4.6. Incidence of PONV

Seventeen RCTs provided numerical data on postoperative nausea and vomiting, while others reported insufficient or zero-event findings [24,29,30,31,37]. Overall, magnesium was associated with a significant reduction in PONV events (OR 0.66, 95% CI 0.44–0.98; *p* = 0.04; I^2^ = 45%).

#### 3.4.7. Patient Satisfaction Scores

Three studies evaluating patient satisfaction (measured on scales ranging from 4-point to 10-point) were included; one RCT was excluded due to the absence of extractable data [29]. The pooled effect size indicated that magnesium improved subjective satisfaction levels (SMD 1.04, 95% CI 0.53–1.56; *p* < 0.0001; I^2^ = 48%).

#### 3.4.8. Intraoperative Remifentanil Consumption

Among the 22 included RCTs, 16 provided analyzable data on the total intraoperative dose of remifentanil, reported either in absolute quantities or converted according to standardized protocols [4,21,23,24,25,26,27,28,31,32,33,34,38,39,40,41]. In the aggregated analysis, magnesium was linked to a significant decrease in remifentanil usage (SMD −0.52, 95% CI −0.86 to −0.18; *p* = 0.003; I^2^ = 85%).

Taken together, these secondary outcomes show that intravenous magnesium does not elevate the risk of hypotension or bradycardia nor does it affect extubation time, yet it appears to reduce the need for rescue analgesia, shivering, and PONV. Additionally, patient satisfaction is higher in those receiving magnesium, and intraoperative remifentanil requirements are lowered, supporting its broader clinical utility under remifentanil-based anesthesia.

### 3.5. Results of Subgroup Analyses

**Figure 7:**
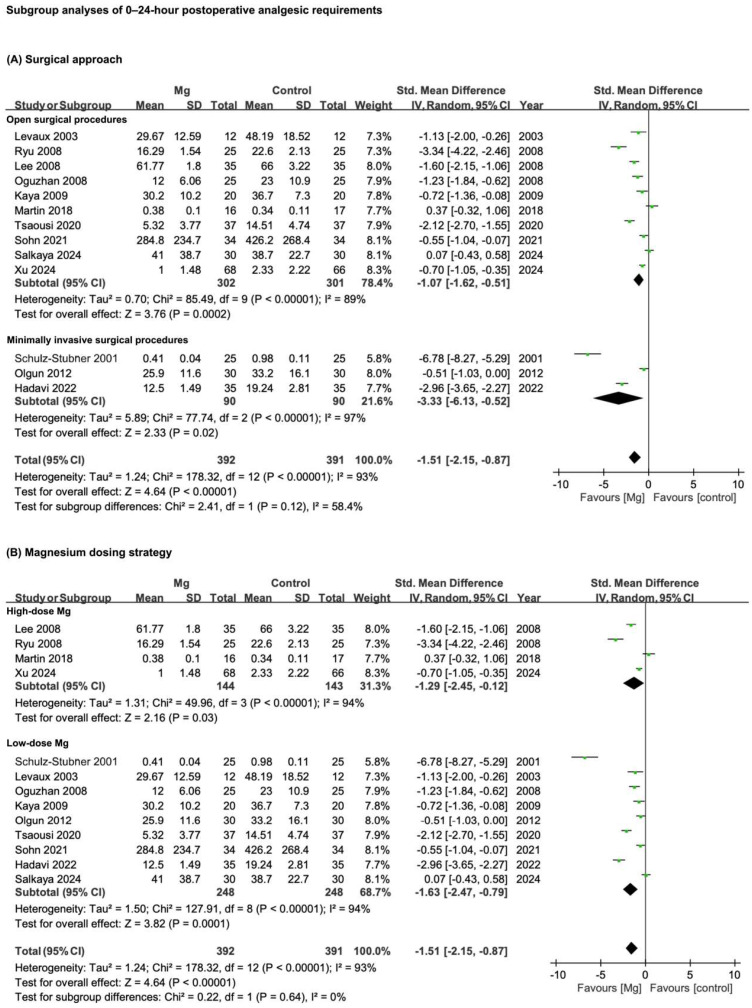
Subgroup analyses of 0–24 h postoperative analgesic requirements. (**A**) Surgical approach (open vs. minimally invasive procedures); (**B**) Magnesium dosing strategy (high-dose vs. low-dose regimens). Effect sizes are expressed as SMDs with 95% CIs, derived from random-effects models. Squares indicate individual study estimates and diamonds represent pooled effects. CI, confidence interval; Mg, magnesium; SMD, standardized mean difference; Std., standard; IV, inverse variance; SD, standard deviation. References [4,21,22,25,26,27,29,31,32,35,36,40,41].

Two pre-specified subgroup analyses were conducted to investigate whether surgical approach (open vs. minimally invasive) or magnesium dosing strategy (high vs. low dose) might account for heterogeneity in the 0–24-h analgesic outcomes. Figure 7 presents the corresponding forest plots.

#### 3.5.1. Surgical Approach: Open vs. Minimally Invasive Procedures

Surgeries were categorized as open or minimally invasive based on anticipated tissue dissection and overall invasiveness. Open procedures included major lumbar orthopedic surgery, cardiac surgery, major abdominal surgery, lumbar disc surgery, total abdominal hysterectomy, lower abdominal laparotomy, posterior spinal fusion for idiopathic scoliosis, lumbar laminectomy, spine surgery, radical mastectomy, orthognathic surgery, and lumbar spine surgery. Minimally invasive interventions comprised pars plana vitrectomy, septorhinoplasty, thyroidectomy, laparoscopic cholecystectomy, robotic radical prostatectomy, and rhinoplasty. Although 14 studies initially appeared to fit the open-surgery criteria, only 10 ultimately provided sufficient data for 0–24 h analgesic requirements [4,22,25,26,27,31,32,35,40,41], while 3 out of 8 nominally “minimally invasive” trials included usable data [21,29,36].

Among the 10 open-surgery trials, magnesium yielded a pooled SMD of −1.07 (95% CI −1.62 to −0.51; *p* = 0.0002; I^2^ = 89%), indicating a pronounced reduction in analgesic requirements. In the three minimally invasive trials, the benefit was similarly significant (SMD −3.33, 95% CI −6.13 to −0.52; *p* = 0.02; I^2^ = 97%); however, the test for subgroup differences (χ^2^ = 2.41, df = 1, *p* = 0.12) was non-significant, suggesting no substantial variance in magnesium’s analgesic-sparing effect between open and minimally invasive procedures.

#### 3.5.2. Magnesium Dosing Strategy

A second subgroup analysis examined whether high-dose or low-dose magnesium administration altered its efficacy in lowering 0–24 h postoperative analgesic requirements. Dosing classifications were based on intravenous bolus and infusion rates reported across the included RCTs (Table 1). Although the literature describes significantly higher regimens (for example, boluses of 50 to 75 mg·kg^−1^ followed by 40 mg·kg^−1^·h^−1^ [42] or a total of 9 g administered over 30 min [43]), adopting these thresholds would have dramatically reduced the number of trials classified as high dose. Furthermore, some evidence suggests that markedly increasing infusion rates may not confer added analgesic benefit but instead heightens hemodynamic concerns [44].

Accordingly, we classified trials with an initial bolus of ≥50 mg·kg^−1^ plus a continuous infusion of ≥10 mg·kg^−1^·h^−1^, or a single bolus of 40 mg·kg^−1^ followed by ≥15 mg·kg^−1^·h^−1^, as “high dose”. Four trials met these criteria [4,25,31,41]. All other protocols were classed as “low dose”. Both categories produced significant reductions in 24 h analgesic requirements (high dose: SMD −1.29, 95% CI −2.45 to −0.12; *p* = 0.03; I^2^ = 94%; low dose: SMD −1.63, 95% CI −2.47 to −0.79; *p* = 0.0001; I^2^ = 94%). The test for subgroup differences (χ^2^ = 0.22, df = 1, *p* = 0.64) indicated that overall effect sizes did not substantially differ between high- and low-dose regimens.

Taken together, these subgroup analyses underscore that magnesium can substantially reduce 24 h analgesic requirements regardless of surgical complexity or dosing strategy. Nevertheless, the high residual heterogeneity (I^2^ ≥ 89%) suggests that other clinical or methodological factors may also shape the degree of benefit.

### 3.6. Sensitivity Analyses

Two predetermined sensitivity analyses were undertaken to gauge the stability of the pooled estimates and to assess whether analytical methods or study inclusion criteria affected the overall conclusions.

#### 3.6.1. Excluding Kilic et al. (2023) [37]

Kilic et al. (2023), rated as high risk of bias, did not provide data on either postoperative analgesic requirements or intraoperative remifentanil usage; hence, excluding this trial was inapplicable to those two endpoints. However, the study reported postoperative pain scores at 0, 12, and 24 h. When these data were removed, the recalculated SMD at 0 h was −0.50 (95% CI −0.79 to −0.21; *p* = 0.0007), −0.95 at 12 h (95% CI −1.72 to −0.17; *p* = 0.02), and −0.67 at 24 h (95% CI −0.95 to −0.38; *p* < 0.00001). In each instance, the analgesic benefit of magnesium remained statistically significant, indicating that exclusion of this high-risk trial did not materially affect our overall conclusions regarding postoperative pain scores.

#### 3.6.2. Re-Analyzing Under a Fixed-Effects Model

To evaluate whether the random-effects approach influenced our findings, we repeated all primary analyses under a fixed-effects (Mantel–Haenszel) framework. The assessment encompassed the following ten endpoints: postoperative analgesic requirements at 0, 6, 24, and 48 h; postoperative pain scores at 0, 6, 12, 24, and 48 h; and overall intraoperative remifentanil consumption.

For postoperative analgesic requirements, the recalculated SMD was −0.74 (95% CI −0.95 to −0.53; *p* < 0.00001) at 0 h, −0.81 (95% CI −1.09 to −0.54; *p* < 0.00001) at 6 h, −1.05 (95% CI −1.21 to −0.89; *p* < 0.00001) at 24 h, and −1.18 (95% CI −1.62 to −0.75; *p* < 0.00001) at 48 h. Similarly, the fixed-effects analysis for postoperative pain scores yielded SMDs of −0.48 (95% CI −0.60 to −0.37; *p* < 0.00001) at 0 h, −0.74 (95% CI −0.92 to −0.56; *p* < 0.00001) at 6 h, −0.41 (95% CI −0.61 to −0.21; *p* < 0.0001) at 12 h, −0.63 (95% CI −0.76 to −0.50; *p* < 0.00001) at 24 h, and −0.41 (95% CI −0.61 to −0.21; *p* < 0.0001) at 48 h, while intraoperative remifentanil consumption also remained in favor of magnesium (SMD −0.55, 95% CI −0.68 to −0.42; *p* < 0.00001).

Across these endpoints, the direction and statistical significance of the effect estimates exhibited only minor deviations from the original random-effects results. Overall, the analgesic advantage of magnesium persisted, with modest shifts in point estimates but no changes in statistical significance. These observations suggest that the modeling approach did not substantially influence the conclusions drawn from our primary meta-analysis.

### 3.7. Publication Bias Analyses Results

**Figure 8:**
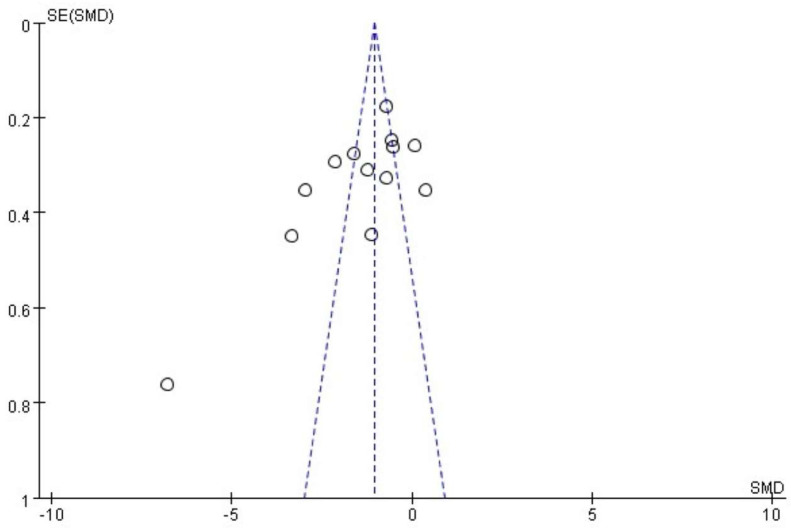
Funnel plot for the meta-analysis of 0–24 h postoperative analgesic requirements with Egger’s test (*p* = 0.02). Each circle denotes an individual RCT, plotted by its SE against the SMD. The vertical dashed line represents the pooled effect estimate and the diagonal dashed lines delineate approximate 95% confidence limits. RCT, randomized controlled trial; SE, standard error; SMD, standardized mean difference.

Because methods such as funnel plots and Egger’s test can be unreliable when fewer than 10 trials are available, we confined our publication bias assessment to outcomes with data from at least 10 RCTs. For 0–24 h analgesic requirements (Figure 8), the funnel plot showed marked asymmetry (Egger’s test, *p* = 0.02), suggesting possible bias. Additionally, we applied Duval and Tweedie’s trim-and-fill procedure for the 0–24 h analgesic outcome (see Supplementary Figure S3). This approach imputed two potentially missing studies, yet the recalculated effect size remained consistently in favor of magnesium, implying that any publication bias might not overturn our primary conclusions. Among the other outcomes that met the same threshold (postoperative pain scores at 24 h, extubation time, incidence of PONV, and intraoperative remifentanil consumption), only extubation time showed a significant Egger’s test result (*p* = 0.03). The remaining three endpoints showed no evidence of publication bias (all *p* > 0.05). Detailed funnel plots and *p*-values are available in Supplementary Figure S2.

### 3.8. GRADE Assessment

Overall certainty of evidence ranged from very low to moderate. Postoperative analgesic requirements (0–24 h) and extubation time both received a “very low” rating, predominantly owing to multiple serious concerns (e.g., risk of bias, inconsistency, or publication bias). In contrast, evidence for intraoperative hypotension, intraoperative bradycardia, patient satisfaction scores, and remifentanil consumption was downgraded to low, reflecting methodological limitations or moderate heterogeneity. By comparison, incidence of shivering and PONV each attained moderate certainty, indicating fewer downgrades. A detailed overview of these findings is provided in the Table 2 alongside the relevant effect estimates.

### 3.9. Additional Subgroup and Meta-Regression Analyses

Although our main analyses revealed persistently high heterogeneity, the initial subgroup evaluations—focused on surgical approach and magnesium dosing—did not substantially reduce it. We therefore conducted additional subgroup analyses for the primary outcome (0–24 h postoperative analgesic requirements), stratifying trials by geographic region, publication year, surgery type, magnesium-administration method, and trial registration status (Supplementary Table S1). While effect sizes varied modestly across these subgroups, the overall heterogeneity remained high, suggesting that none of these categorical factors alone accounted for the between-study variability.

To further explore potential moderating influences, we performed a meta-regression on the same outcome using the following four quantitatively coded variables: mean age, percentage of female participants, total sample size, and ASA classification. Although each moderator was analyzed separately (Supplementary Table S2), no significant relationship emerged between any variable and the effect estimate, and heterogeneity remained elevated.

## 4. Discussion

In this meta-analysis of 22 randomized controlled trials, we observed that the adjunctive use of intravenous magnesium in remifentanil-based anesthesia led to significantly lower analgesic requirements and reduced pain intensity within the initial 24 h postoperatively. Although patients in the magnesium arm continued to demonstrate improved pain scores up to 48 h, analgesic requirements in both groups converged to a similar level by that time. This convergence could be attributed to the fact that most participants reached generally acceptable pain thresholds, and so incremental improvements in pain scores did not necessarily translate into elevated analgesic requirements. Moreover, the consistency of these findings—verified through sensitivity analyses and under a fixed-effects model—underscores the robustness of our conclusions despite potential methodological or patient-related heterogeneity. Despite variability in effect sizes across individual trials, the broader evidence base supports magnesium as a favorable adjunct to remifentanil protocols.

In addition to these primary outcomes, various secondary endpoints were assessed, namely intraoperative hypotension and bradycardia, extubation time, rescue-analgesia requirements, shivering, PONV, patient satisfaction, and intraoperative remifentanil usage. Corroborating our main analyses, magnesium infusion did not notably raise the incidence of hypotension or bradycardia, thus minimizing concerns over hemodynamic safety [45]. Furthermore, extubation times remained comparable between treatment arms, suggesting that magnesium did not adversely affect emergence or overall recovery trajectories. By contrast, participants receiving magnesium exhibited substantially decreased rescue-analgesic requirements, consistent with their lower pain scores and diminished opioid requirements over the initial 24 h. Improved satisfaction ratings, less shivering, fewer PONV events, and reduced total remifentanil consumption collectively highlight magnesium’s multi-pronged benefits in this setting.

From a mechanistic standpoint, magnesium is believed to inhibit the excessive neuronal hyperexcitability triggered by remifentanil partly via NMDA receptor blockade. Excessive NMDA receptor activation can also drive p38 MAPK, a key modulator of neuroinflammatory changes in OIH [3]. Experimental studies indicate that magnesium may restore or reinforce the voltage-dependent Mg^2+^ block of the NMDA channel, curtailing pathologically enhanced calcium influx and downstream pro-nociceptive signaling [5]. Some data also hint at magnesium’s capacity to modulate neuroinflammatory responses and glial activation [46], although robust clinical trials linking these cellular events to specific analgesic outcomes remain lacking. Notwithstanding these biological premises, certain reviews have reported inconclusive effects, potentially attributable to variation in magnesium dose regimens, patient demographics, or surgical settings [8,9]. In addition, some systematic reviews have targeted only restricted populations, such as spinal surgery [10,47], or addressed other analgesic modulators alongside magnesium [48,49]. By contrast, our analysis encompassed a broad range of procedures yet specifically focused on magnesium supplementation in remifentanil-based anesthesia, offering an inclusive yet more uniform viewpoint. The subgroup comparisons (high-dose vs. low-dose magnesium and open vs. minimally invasive surgery) further illuminate the flexible clinical applicability of magnesium in diverse operative contexts. Nevertheless, these encouraging results have not always been mirrored in earlier meta-analyses.

Despite the growing body of evidence, previous meta-analyses have sometimes reached divergent conclusions regarding magnesium’s role in remifentanil-induced hyperalgesia. Several factors may account for these discrepancies including small sample sizes, variations in patient populations, and inconsistent outcome assessments. Publication bias could also play a part if studies with neutral or unfavorable results were less likely to be published. Recognizing such limitations highlights the need to interpret earlier findings with caution and underscores the importance of well-designed, large-scale trials that use standardized protocols. A further challenge lies in the diverse ways magnesium has been administered across these studies. Regimens range from modest bolus injections to sizeable loading doses followed by prolonged infusions, with no consensus on the most effective or safest strategy. Additionally, we examined whether the route and timing of magnesium administration (bolus-only vs. bolus followed by continuous infusion) might influence outcomes. Although the bolus-only subset appeared to exhibit a somewhat larger effect size (SMD −3.54; 95% CI −6.06 to −1.03) compared with the bolus-plus-infusion approach (SMD −1.13; 95% CI −1.68 to −0.57), the heterogeneity remained substantial in both groups (see Supplementary Table S1). With only a small number of trials contributing to each subgroup, these results warrant cautious interpretation. Future studies adopting more uniform magnesium administration protocols may help elucidate whether infusion strategies materially alter the analgesic advantage. In our subgroup comparisons, we attempted to classify magnesium use as higher or lower dose, yet these analyses did not yield a single best regimen. These inconsistencies emphasize the value of future research with more uniform dosing schedules to identify a dose–response relationship and, ultimately, the optimal magnesium protocol. Hence, clarifying these aspects becomes crucial for determining magnesium’s genuine role in remifentanil-induced hyperalgesia.

In practical terms, these findings align with the concept of multimodal analgesia, wherein multiple interventions collectively diminish reliance on a single pharmacological modality [50]. We noted that adjunctive magnesium not only reduces opioid dosage but may also mitigate OIH. Earlier meta-analyses occasionally failed to demonstrate a significant reduction in PONV [8,9,48,49], yet subsequent investigations focusing on spine surgery populations [10,47] reported tangible decreases in emetic events—a trend mirrored by our broader dataset. Likewise, magnesium has been linked to a prolonged interval before the first postoperative analgesia request, while neither the time to follow commands nor extubation duration appear significantly affected, underscoring a potential synergy among analgesic efficacy, opioid sparing, and patient comfort (Supplementary Figure S1).

A number of prior investigations have advocated high-dose magnesium administration for optimal pain relief [42], whereas other reports suggest that lower infusion regimens may also impart meaningful benefits [43,44]. Our subgroup analyses did not reveal major differentials between high- and low-dose magnesium in reducing analgesic requirements, implying that clinicians may individualize regimens based on patient characteristics or procedural demands. Moreover, the degree of surgical invasiveness (open vs. minimally invasive) did not significantly alter magnesium’s analgesic effect, although moderate or high I^2^ values suggest marked heterogeneity, warranting cautious interpretation. Such variability may stem from differences in patient populations, anesthetic regimens, outcome measurements, or surgical complexity, highlighting the need for more standardized methodologies in future trials. This high heterogeneity likely reflects variation in patient comorbidities, procedural complexity, and dosing protocols, which further supports the need for individualized approaches in daily practice. Follow-up studies incorporating real-time nociceptive monitoring or tailored dosing algorithms could refine the role of magnesium in daily practice. Nevertheless, clinicians should remain aware of absolute or relative contraindications, including severe renal dysfunction, hemodynamic instability, or neuromuscular junction disorders such as myasthenia gravis.

Our sensitivity analyses bolstered confidence in these conclusions. Excluding one high-risk trial did not diminish the analgesic-sparing effect of magnesium, and employing a fixed-effects model yielded results consistent with the random-effects estimations. These findings reduce the likelihood that any single study or specific statistical assumption drove our primary outcomes. However, application of GRADE revealed that many endpoints were hampered by “some concerns”, small sample sizes, and inconsistent measurement methods. Furthermore, unreported negative or neutral trials may have inflated magnesium’s apparent benefit. Larger-scale randomized studies with standardized protocols, extended follow-up, and clinically meaningful endpoints are needed to determine the minimal effective magnesium dose compare bolus-only vs. infusion strategies and clarify safety margins in higher-risk patients. Monitoring serum magnesium levels or adopting dose-standardization practices may be advantageous, particularly in individuals with significant cardiac comorbidities, to ensure both efficacy and safety.

Additionally, several included RCTs specifically addressed serum magnesium concentrations and potential hypermagnesemia-related events albeit to varying degrees. For instance, Olgun et al. (2012) [29] reported four patients requiring postoperative reintubation, which they partially attributed to magnesium’s enhancement of neuromuscular blockade, although residual muscle relaxants could not be fully excluded. Steinlechner et al. (2006) [23] cautioned that levels above 2.5 mmol/L may increase neuromuscular risks but did not document overt clinical complications, whereas Salkaya et al. (2024) [40] observed moderate serum magnesium elevations without any significant adverse outcomes. Collectively, these findings highlight that while severe hypermagnesemia is uncommon in standard dosing regimens, underreporting remains possible, especially when neuromuscular monitoring is lacking. Future investigations with rigorous serum magnesium surveillance and standardized neuromuscular assessment will help clarify the true incidence and impact of this issue.

Moreover, although these findings underscore magnesium’s promise as an adjunct in remifentanil-based anesthesia, anesthesiologists should balance its benefits against patient-specific considerations such as neuromuscular disorders and overall comorbidity. Tailoring the magnesium regimen accordingly may help optimize analgesic efficacy while minimizing potential adverse effects in routine practice. Clinically, an intravenous bolus of about 30 to 50 mg·kg^−1^ followed by a maintenance infusion of 10 to 15 mg·kg^−1^·h^−1^ is often recommended to achieve adequate analgesic effects without excessive hemodynamic compromise [4,42,43,44]. However, real-world experience shows a wide variation in dosing strategies and patient responses, emphasizing the importance of individualized care. Measuring serum magnesium can be prudent, especially for prolonged infusions or in patients with borderline renal function. Ultimately, future studies incorporating advanced nociceptive monitoring and mechanistic biomarkers, such as neuroinflammatory markers or quantitative sensory testing, may refine magnesium’s therapeutic window in remifentanil-based anesthesia and further alleviate the burden of OIH.

### Limitations

Several limitations of this meta-analysis warrant mention. First, most included trials were rated “some concerns” in RoB 2, reflecting potential methodological or reporting issues that may undermine internal validity. Second, marked heterogeneity was evident across studies in terms of surgical approach, magnesium dosing, and outcome measures, possibly restricting the precision of pooled estimates. None of our additional subgroup or meta-regression analyses substantially reduced heterogeneity, implying that certain unmeasured or unreported clinical and methodological variables remain. This observation underscores the need for more standardized protocols and detailed patient-level data in future research so that magnesium’s role in remifentanil-based anesthesia can be delineated more clearly. Third, funnel-plot analyses and the potential omission of unpublished or grey literature both suggest an overestimation of the positive effects. Lastly, the limited availability of large, high-quality RCTs constrains the generalizability of our findings. We therefore emphasize that more robust, large-scale RCTs with standardized protocols, consistent analgesic strategies, and thorough patient-level data collection are needed. Such efforts would allow a clearer understanding of magnesium’s optimal use in remifentanil-based anesthesia while mitigating the persistently high heterogeneity observed.

## 5. Conclusions

This meta-analysis of 22 randomized controlled trials demonstrates that intravenous magnesium as an adjunct to remifentanil-based anesthesia significantly reduces postoperative analgesic requirements and pain intensity, particularly within the first 24 h, with effects persisting up to 48 h. In addition, magnesium lowers the incidence of shivering, postoperative nausea and vomiting, and intraoperative remifentanil consumption without prolonging extubation or increasing the risk of hypotension and bradycardia. Nonetheless, the evidence quality—rated moderate to very low—along with high heterogeneity and potential publication bias calls for caution in interpreting these results. Future large-scale trials with standardized protocols and comprehensive serum monitoring are essential to better define optimal dosing, safety profiles, and magnesium’s role in mitigating remifentanil-induced hyperalgesia. Overall, these findings support intravenous magnesium as a promising, adaptable adjunct to enhance perioperative pain control in diverse surgical settings.

## Figures and Tables

**Table 1:** Characteristics of the RCTs included in the meta-analysis.

Study (Author/Year)	Country	Participants	Gender (M/F)	Mean Age ± SD	ASA	Type of Surgery	Magnesium Intervention	Remifentanil Infusion Rate	Premedication	Anaesthesia Maintenance	Postoperative Analgesia	Trial Registration ID
Schulz-Stübner 2001 [21]	Germany	Mg: 25 Control: 25	Mg: 12/13 Control: 15/10	Mg: 60.76 ± 14.9 Control: 51.76 ± 17.7	I–III	Pars plana vitrectomy	Magnesium sulfate 50 mg kg^−1^ IV	0.1 mcg kg^−1^min^−1^ (both groups)	Midazolam 3.75–7.5 mg orally	Propofol Remifentanil Mivacurium	Metamizol 10 mg kg^−1^ IV + Nalbuphine for VRS > 3	Not reported
Levaux 2003 [22]	Belgium	Mg: 12 Control: 12	Mg: 4/8 Control: 7/5	Mg: 55 ± 16 Control: 46 ± 19	I–II	Major lumbar orthopedic surgery	Magnesium sulfate 50 mg kg^−1^ IV	0.25 mcg kg^−1^min^−1^ (both groups)	Alprazolam 1 mg Atropine 0.5 mg orally	Sevoflurane Remifentanil Nitrous oxide Rocuronium	PCA with piritramide	Not reported
Steinlechner 2006 [23]	Austria	Mg: 19 Control: 20	Mg: 15/4 Control: 16/4	Mg: 59 (23–79) Control: 61 (33–77) * Mean (range)	I–IV	Cardiac surgery	Magnesium gluconate 86.5 mg kg^−1^ IV followed by continuous infusion of 13.8 mg kg^−1^h^−1^ for 12 h after extubation	0.15–0.5 mcg kg^−1^min^−1^ (both groups)	Midazolam 7.5 mg orally	Sevoflurane Remifentanil Cisatracurium	Remifentanil infusion (titrated) followed by piritramide and paracetamol	Not reported
Cizmeci 2007 [24]	Turkey	Mg: 30 Control: 30	Mg: 10/20 Control: 12/18	Mg: 25.8 ± 7.6 Control: 26.5 ± 7.9	I–II	Septorhinoplasty	Magnesium sulfate 50 mg kg^−1^ IV + 8 mg kg^−1^h^−1^ continuous infusion	0.1 mcg kg^−1^min^−1^ (both groups)	None	Propofol Remifentanil Vecuronium	Meperidine 1 mg kg^−1^ IM for VRS > 4	Not reported
Lee 2008 [25]	South Korea	Mg: 35 Control: 35	Mg: 16/19 Control: 18/17	Mg: 62.1 ± 3.8 Control: 61.4 ± 4.2	I–II	Major abdominal surgery	Magnesium sulfate 50 mg kg^−1^ IV + 10 mg kg^−1^h^−1^ continuous infusion	0.25 mcg kg^−1^min^−1^ (both groups)	None	Sevoflurane Remifentanil Rocuronium	PCA with morphine and ketorolac	Not reported
Oguzhan 2008 [26]	Turkey	Mg: 25 Control: 25	Mg: 13/12 Control: 14/11	Mg: 44 (41–48) Control: 42 (38–46) * Mean (95%CI)	I–II	Lumbar disc surgery	Magnesium sulfate 30 mg kg^−1^ IV + 10 mg kg^−1^h^−1^ continuous infusion	1.67 mcg kg^−1^min^−1^ (both groups)	Not reported	Sevoflurane Remifentanil Nitrous oxide Atracurium	PCA with morphine	Not reported
Ryu 2008 [4]	South Korea	Mg: 25 Control: 25	All female	Mg: 41.1 Control: 43.7	I–II	Total abdominal hysterectomy	Magnesium sulfate 50 mg kg^−1^ IV + 15 mg kg^−1^h^−1^ continuous infusion	TCI 4.0 ng mL^−1^ (both groups)	Not reported	Propofol Remifentanil Rocuronium	PCA with morphine and ketorolac	Not reported
Kaya 2009 [27]	Turkey	Mg: 20 Control: 20	All female	Mg: 50 ± 10.7 Control: 50 ± 7.5	I–II	Total abdominal hysterectomy	Magnesium sulfate 30 mg kg^−1^ IV + 500 mg h^−1^ continuous infusion	0.25 mcg kg^−1^min^−1^ (both groups)	Diazepam 10 mg orally	Sevoflurane Remifentanil Cisatracurium	PCA with morphine	Not reported
Song 2011 [28]	South Korea	Mg: 28 HI: 28 (High-dose remifentanil) LO: 28 (Low-dose remifentanil)	Mg: 6/22 HI: 5/23 LO: 4/24	Mg: 45 ± 10 HI: 47 ± 11 LO: 47 ± 10	I–II	Thyroidectomy	Magnesium sulfate 30 mg kg^−1^ IV + 10 mg kg^−1^h^−1^ continuous infusion	Mg/HI: 0.20 mcg kg^−1^min^−1^ LO: 0.05 mcg kg^−1^min^−1^	Midazolam 0.05 mg kg^−1^ IM	Sevoflurane Remifentanil Rocuronium	PACU: Fentanyl IV for VNRS > 4 General ward: Tramadol 37.5 mg + acetaminophen 325 mg orally	NCT01025245
Olgun 2012 [29]	Turkey	Mg: 30 Control: 30	Mg: 5/25 Control: 5/25	Mg: 47.7 ± 11.4 Control: 45.4 ± 12.4	I–II	Laparoscopic cholecystectomy	Magnesium sulfate 40 mg kg^−1^ IV + 10 mg kg^−1^h^−1^ continuous infusion	0.25 mcg kg^−1^min^−1^ to 0.125 mcg kg^−1^min^−1^ 10 min after intubation (both groups)	Midazolam 0.07 mg kg^−1^ IM	Desflurane Nitrous oxide Remifentanil Vecuronium	PCA with morphine	Not reported
Asadollah 2015 [30]	Iran	Mg: 15 Control: 15	All female	Mg: 48.6 ± 4.91 Control: 49.1 ± 4.8	I–II	Lower abdominal laparotomy	Magnesium sulfate 50 mg kg^−1^ IV + 8 mg kg^−1^h^−1^ continuous infusion	0.40 mcg kg^−1^min^−1^ (both groups)	Midazolam 0.2 mg kg^−1^ IV	Propofol Remifentanil Atracurium	Fentanyl 1 mcg kg^−1^ + Meperidine 30 mg IV for VRS > 4	Not reported
Martin 2018 [31]	United States	Mg: 19 (Remifentanil + Mg) MET: 22 (Remifentanil + methadone) REMI: 19 (Remifentanil alone)	Mg: 3/16 MET: 5/17 REMI: 3/16	Mg: 15.3 ± 1.9 MET: 15.4 ± 1.2 REMI: 14.2 ± 1.4	I–III	Posterior spinal fusion for idiopathic scoliosis	Mg: Magnesium sulfate 50 mg kg^−1^ IV + 10 mg kg^−1^h^−1^ continuous infusion	0.05–0.3 mcg kg^−1^min^−1^ (all groups)	Midazolam 20 mg orally	Desflurane Nitrous oxide Remifentanil Rocuronium	PCA with hydromorphone + acetaminophen orally and ketorolac IV	NCT01795495
Tsaousi 2020 [32]	Greece	Mg: 35 Control: 36	Mg: 13/22 Control: 15/21	Mg: 55.9 ± 10.8 Control: 49.0 ± 15.0	I–III	Lumbar laminectomy	Magnesium sulfate 20 mg kg^−1^ IV + 20 mg kg^−1^h^−1^ continuous infusion	TCI mode: Plasma concentration 1.5–3.0 ng mL^−1^	Diazepam 5–10 mg orally	Desflurane Remifentanil Cisatracurium	Paracetamol 1 g IV Lornoxicam 8 mg orally Morphine 3 mg IV	NCT04161729
Kim 2021 [33]	South Korea	Mg: 26 Control: 26	All male	Mg: 63 ± 7 Control: 65 ± 7	I–II	Robotic radical prostatectomy	Magnesium sulfate 50 mg kg^−1^ IV + 10 mg kg^−1^h^−1^ continuous infusion	TCI 0.5–4.0 ng mL^−1^	Not reported	Sevoflurane Remifentanil Rocuronium	PCA with fentanyl + nefopam and ibuprofen IV + acetaminophen orally	NCT02833038
Lu 2021 [34]	China	Mg: 37 Lidocaine: 37 Control: 40	Mg: 8/29 Lidocaine: 8/29 Control: 11/29	Mg: 46.8 ± 13.3 Lidocaine: 42.2 ± 10.5 Control: 45.4 ± 11.7	I–II	Laparoscopic cholecystectomy	Magnesium sulfate 20 mg kg^−1^ IV + 20 mg kg^−1^h^−1^ continuous infusion	TCI 4.7–8 ng mL^−1^	Not reported	Propofol Remifentanil Cisatracurium	Flurbiprofen 100 mg IV + Fentanyl 50 mcg IV for NRS > 3	ChiCTR1800019092
Sohn 2021 [35]	South Korea	Mg: 34 Control: 34	Mg:14/20 Control: 17/17	Mg: 56.5 ± 13.7 Control: 56.5 ± 14.7	I–III	Spine surgery	Magnesium sulfate 30 mg kg^−1^ IV + 15 mg kg^−1^h^−1^ continuous infusion	TCI 1.0–5.0 ng mL^−1^	Not reported	Propofol Remifentanil Rocuronium	PCA with fentanyl + rescue analgesics depending on patients’ need (morphine, propacetamol, meperidine, fentanyl, acetaminophen, ketorolac, and nefopam)	KCT0004173
Hadavi 2022 [36]	Iran	Mg: 35 Pregabalin: 35 Control: 35	Mg: 8/27 Pregabalin: 4/31 Control: 12/23	Mg: 29.31 ± 7.94 Pregabalin: 28.12 ± 6.57 Control: 25.47 ± 4.81	Not reported	Rhinoplasty	Magnesium sulfate 30 mg kg^−1^ IV	0.3 mcg kg^−1^min^−1^ (All groups)	Not reported	Isoflurane Nitrous oxide Remifentanil Atracurium	Morphine 1-2 mg every 5 min until NRS < 4	IRCT20121204011662N12
Kilic 2023 [37]	Turkey	Mg: 60 Control: 60	Mg:20/40 Control: 17/43	Ages 18–45 (both groups)	Not reported	Septorhinoplasty	Magnesium sulfate 30 mg kg^−1^ IV + 9 mg kg^−1^h^−1^ continuous infusion	0.25–1 mcg kg^−1^min^−1^ (both groups)	Not reported	Sevoflurane Remifentanil Rocuronium	Dexketoprofen 25 mg IV + Tramadol 1 mg kg^−1^ IV for NRS > 4	Not reported
Su 2023 [38]	China	Mg: 35 Control: 35	All female	Mg: 52 (48–57) Control: 54 (48–59) * Median (range)	I–II	Radical mastectomy	Magnesium sulfate 30 mg kg^−1^ IV + 10 mg kg^−1^h^−1^ continuous infusion	0.1 mcg kg^−1^min^−1^ (both groups)	None	Sevoflurane Remifentanil Cisatracurium	Fentanyl 1 mcg kg^−1^ + Tramadol 25 mg IV for emergency agitation	ChiCTR2300070595
Hua 2024 [39]	China	Mg: 36 Control: 37	Mg:12/24 Control: 13/24	Mg: 24 ± 4 Control: 24 ± 5	I–II	Orthognathic surgery	Magnesium sulfate 50 mg kg^−1^ IV + 15 mg kg^−1^h^−1^ continuous infusion	0.1 mcg kg^−1^min^−1^ (both groups)	None	Sevoflurane Dexmedetomidine Remifentanil Cisatracurium	PACU: Fentanyl 20-30 mcg IV for NRS > 3 Ward: Celecoxib orally + tramadol IM	ChiCTR2100045981
Salkaya 2024 [40]	Turkey	Mg: 30 Control: 30	Mg:13/17 Control: 12/18	Mg: 58.4 ± 9.5 (range 44–80) Control: 60.3 ± 11 (range 36–82)	I–II	Lumbar spine surgery	Magnesium sulfate 10 mg kg^−1^h^−1^ continuous infusion	Not reported	None	Sevoflurane Remifentanil Rocuronium	PCA with meperidine + diclofenac sodium for VAS > 4	Not reported
Xu 2024 [41]	China	Mg: 68 Control: 66	Mg:14/54 Control: 16/50	Mg: 67.00 (64.00–70.00) Control: 66.00 (62.00–71.00) * Median (IQR)	II–III	Total knee arthroplasty	Magnesium sulfate 40 mg kg^−1^ IV + 15 mg kg^−1^h^−1^ continuous infusion	0.1–0.3 mcg kg^−1^min^−1^ (both groups)	Not reported	Propofol Remifentanil Cisatracurium	PCA with fentanyl + femoral nerve block with 20 mL of 0.25% ropivacaine	ChiCTR2200065940

* The RCT did not report age data as mean ± SD. RCTs, randomized controlled trials; Mg, magnesium; HI, high-dose remifentanil; LO, low-dose remifentanil; SD, standard deviation; CI, confidence interval; ASA, American Society of Anesthesiologists Physical Status classification; IV, intravenous; TCI, target-controlled infusion; IM, intramuscular; VRS, verbal rating scale; PCA, patient-controlled analgesia; VNRS, verbal numeric rating scale; MET, remifentanil + methadone; REMI, remifentanil alone; IQR, interquartile range; NRS, numeric rating scale; VAS, visual analogue scale.

**Table 2:** GRADE summary of findings for key outcomes.

Certainty Assessment											
Outcome	No. of Trials	Study Design	Risk of Bias	Inconsistency	Indirectness	Imprecision	Other	No. of Patients (n)		Effect Estimate (95% CI)	Quality of Evidence
								Mg	Control		
Postoperative analgesic requirements—0–24 h	13	RCTs	Serious *	Serious **	Not serious	Not serious	Publication bias	393	392	SMD −1.51 (−2.15 to −0.87)	⊕○○○
											Very low
Postoperative pain scores—24 h	14	RCTs	Serious *	Serious **	Not serious	Not serious	None	488	491	SMD −0.61 (−0.90 to −0.32)	♁♁○○
											Low
Intraoperative hypotension incidence	7	RCTs	Serious *	Not serious	Not serious	Serious ***	None	193	197	SMD 1.44 (0.92 to 2.26)	♁♁○○
											Low
Intraoperative bradycardia incidence	8	RCTs	Serious *	Not serious	Not serious	Serious ***	None	287	290	SMD 1.31 (0.67 to 2.57)	♁♁○○
											Low
Extubation time	14	RCTs	Serious *	Not serious	Not serious	Serious ***	Publication bias	444	447	SMD −0.04 (−0.18 to 0.11)	♁○○○
											Very low
Number of patients requiring rescue analgesia	8	RCTs	Serious *	Serious **	Not serious	Not serious	None	253	251	SMD 0.32 (0.15 to 0.70)	♁♁○○
											Low
Incidence of shivering	8	RCTs	Serious *	Not serious	Not serious	Not serious	None	248	248	SMD 0.25 (0.12 to 0.52)	♁♁♁○
											Moderate
Incidence of PONV	17	RCTs	Serious *	Not serious	Not serious	Not serious	None	525	529	SMD 0.66 (0.44 to 0.98)	♁♁♁○
											Moderate
Patient satisfaction scores	3	RCTs	Serious *	Not serious	Not serious	Serious ***	None	72	73	SMD 1.04 (0.53 to 1.56)	♁♁○○
											Low
Intraoperative remifentanil consumption	16	RCTs	Serious *	Serious **	Not serious	Not serious	None	493	497	SMD −0.52 (−0.86 to −0.18)	♁♁○○
											Low

GRADE, grading of recommendations, assessment, development, and evaluation; No., number; Mg, magnesium; RCT, randomized controlled trial; CI, confidence interval; SMD, standard mean difference; PONV, postoperative nausea and vomiting. * The included studies, evaluated using the RoB2 tool, were judged to have either some concerns or a high risk of bias. ** Substantial heterogeneity (I^2^ > 50%). *** 95% Confidence interval includes both magnesium and control (*p* > 0.05).

## Data Availability

All data are contained in the article.

## Supplementary Materials

The following supporting information can be downloaded at: https://www.mdpi.com/article/10.3390/ph18040518/s1. Figure S1. Forest plots showing two additional outcomes comparing Mg with control: (A) Time to first analgesic and; (B) Time to follow commands. Effect sizes are reported as SMDs with 95% CIs, derived from a random-effects model using the IV method. CI, confidence interval; IV, inverse variance; Mg, magnesium; SD, standard deviation; SMD, standardized mean difference; Std., standard. Figure S2. Funnel plots for the meta-analyses of four outcomes with at least ten RCTs each: (A) postoperative pain scores at 24 h (14 RCTs; Egger’s test, *p* = 0.71); (B) Extubation time (14 RCTs; Egger’s test, *p* = 0.03); (C) Incidence of PONV (17 RCTs; Egger’s test, *p* = 0.36); (D) Intraoperative remifentanil consumption (16 RCTs; Egger’s test, *p* = 0.45). Each open circle represents an individual RCT, plotted by its SE on the vertical axis against the effect estimate—either the SMD or the logarithm of the OR—on the horizontal axis. The vertical solid line indicates the pooled effect estimate, and the diagonal dashed lines depict approximate 95% confidence limits. RCT, randomized controlled trial; SE, standard error; SMD, standardized mean difference; PONV, postoperative nausea and vomiting; OR, odds ratio. Figure S3. Duval and Tweedie’s Trim-and-Fill funnel plot for 0–24-h postoperative analgesic requirements. The open circles represent the original observed studies, and the black circles denote two imputed (i.e., “missing”) studies to the left of the funnel’s mean effect. After imputing these additional points, the random-effects summary estimate shifted from an SMD of approximately −1.5 to −1.9, indicating that the true analgesic benefit of magnesium might be slightly stronger than initially observed. Nevertheless, the overall direction and statistical significance of the effect remained consistent. Std diff, standardized difference; SMD, standardized mean difference. Table S1. Additional subgroup analyses for 0–24-h postoperative analgesic requirements. Table S2. Meta-regression analyses for 0–24-h postoperative analgesic requirements.

## Abbreviations

The following abbreviations are used in this manuscript:

OIH	Opioid-induced hyperalgesia
NMDA	N-methyl-D-aspartate
MAPK	Mitogen-activated protein kinase
RCT	Randomized controlled trial
SD	Standard deviation
SE	Standard error
CI	Confidence interval
IQR	Interquartile range
PONV	Postoperative nausea and vomiting
RoB	Risk of bias
GRADE	Grading of recommendations, assessment, development, and evaluation
PCA	Patient-controlled analgesia
VAS	Visual analogue scale
SMD	Standardized mean difference
OR	Odds ratio
ASA	American Society of Anesthesiologists
NAS	Numeric analogue scale
NRS	Numeric rating scale
VNRS	Verbal numeric rating scale
TCI	Target-controlled infusion
