# Supplementary material for: Pharmacological Efficacy of Intravenous Magnesium in Attenuating Remifentanil-Induced Postoperative Hyperalgesia: A Systematic Review and Meta-Analysis of Randomized Controlled Trials
Source: Pharmaceuticals (Basel). 2025 Apr 1;18(4):518. doi: 10.3390/ph18040518 (PMC12030599; doi:10.3390/ph18040518)
Supplement: Supplementary file 1 [file pharmaceuticals-18-00518-s001.zip › pharmaceuticals-3537290-Figures and tables.docx]

**Supplementary Materials**


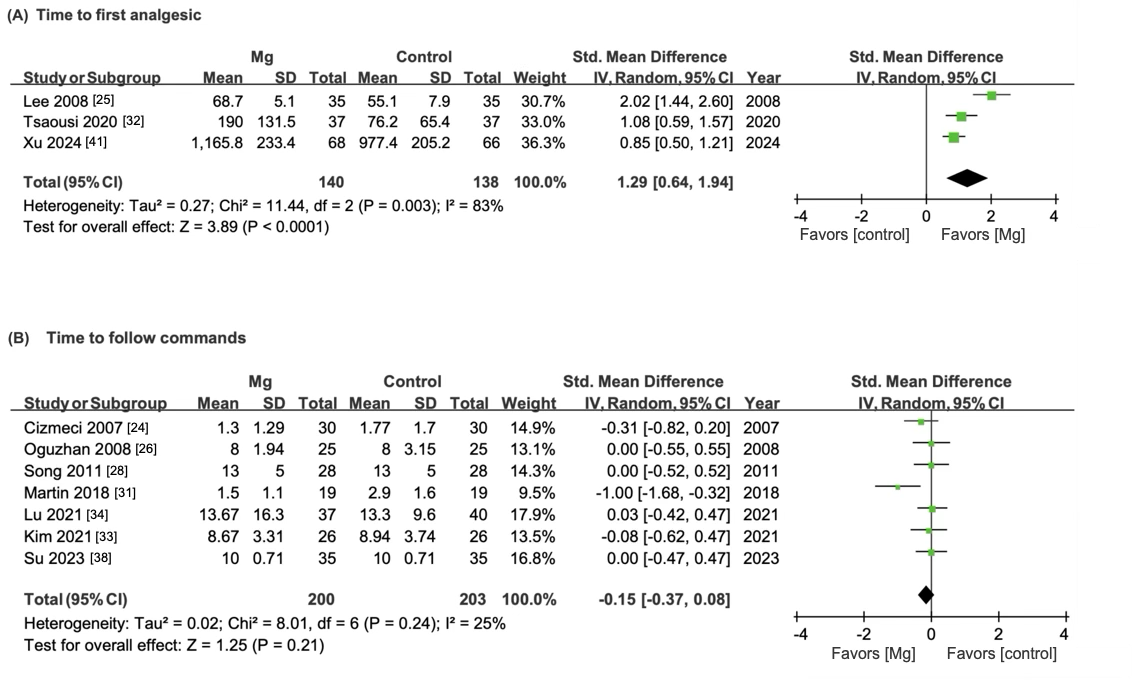


**Figure S1.** Forest plots showing two additional outcomes comparing Mg with control: (**A**) Time to first analgesic and; (**B**) Time to follow commands. Effect sizes are reported as SMDs with 95% CIs, derived from a random-effects model using the IV method. CI, confidence interval; IV, inverse variance; Mg, magnesium; SD, standard deviation; SMD, standardized mean difference; Std., standard.


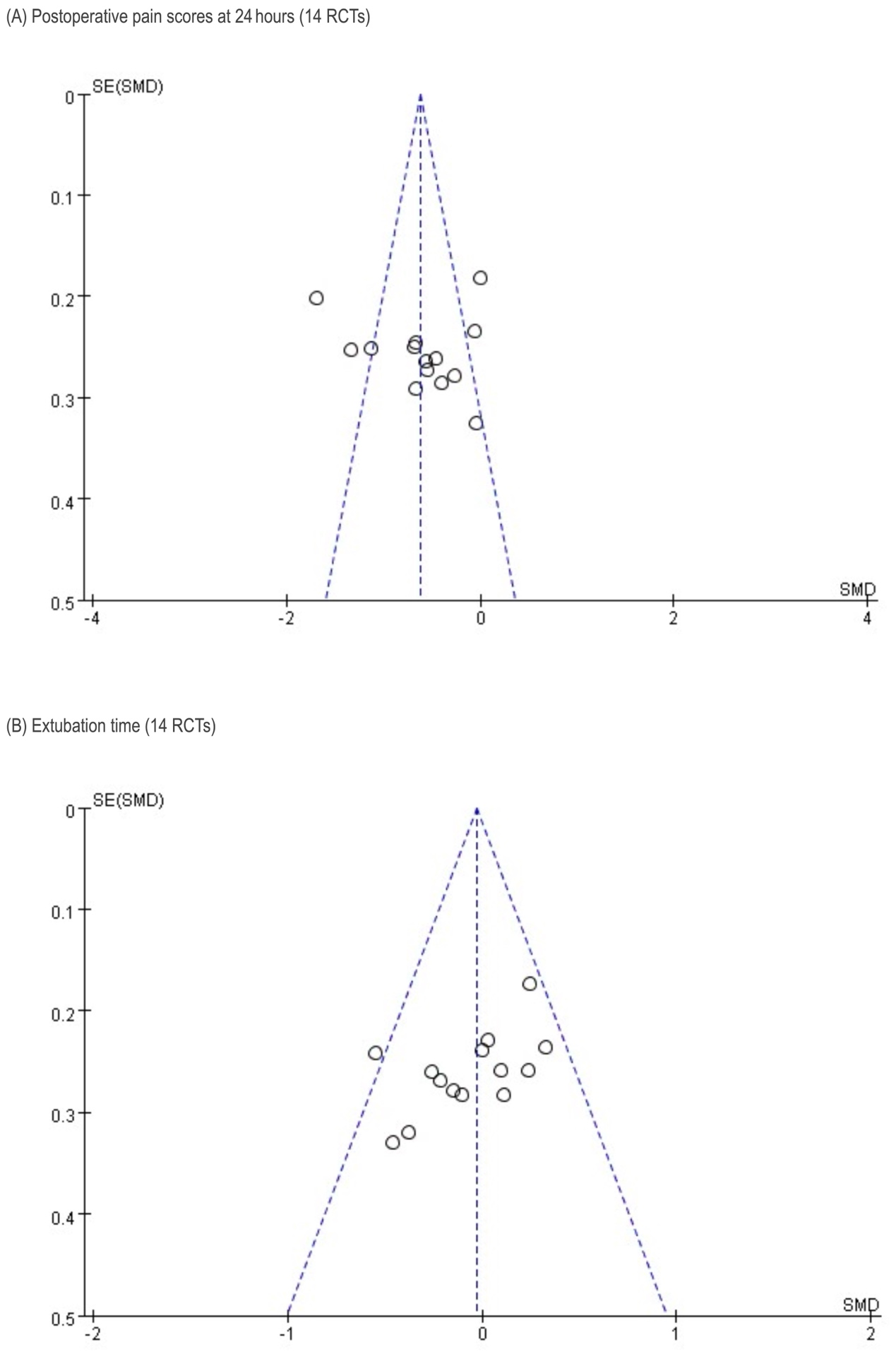


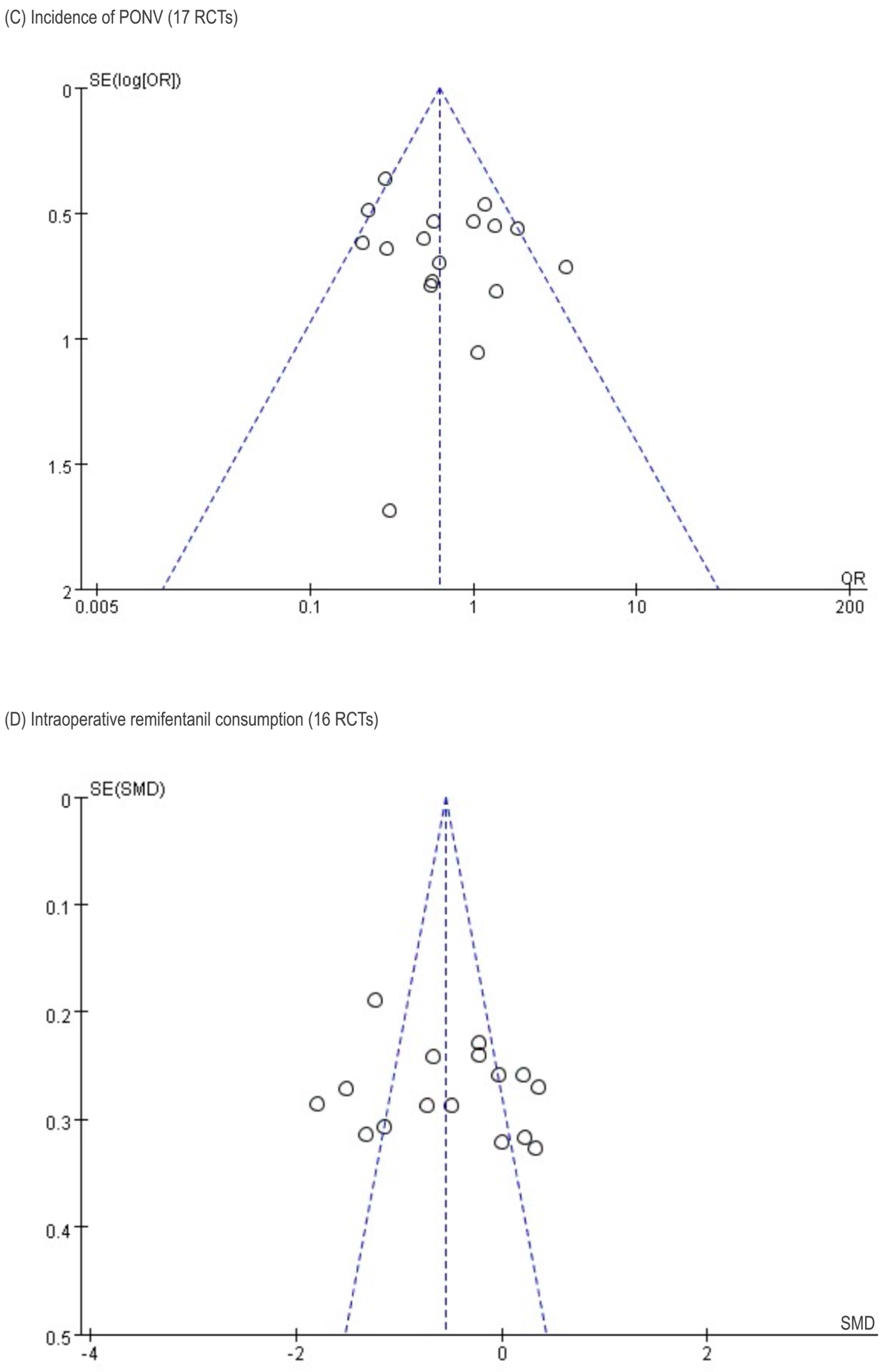


**Figure S2.** Funnel plots for the meta‐analyses of four outcomes with at least ten RCTs each: (**A**) postoperative pain scores at 24 hours (14 RCTs; Egger’s test, *p*= 0.71); (**B**) Extubation time (14 RCTs; Egger’s test, *p*= 0.03); (**C**) Incidence of PONV (17 RCTs; Egger’s test, *p*= 0.36); (**D**) Intraoperative remifentanil consumption (16 RCTs; Egger’s test, *p*= 0.45). Each open circle represents an individual RCT, plotted by its SE on the vertical axis against the effect estimate—either the SMD or the logarithm of the OR—on the horizontal axis. The vertical solid line indicates the pooled effect estimate, and the diagonal dashed lines depict approximate 95% confidence limits. RCT, randomized controlled trial; SE, standard error; SMD, standardized mean difference; PONV, postoperative nausea and vomiting; OR, odds ratio.


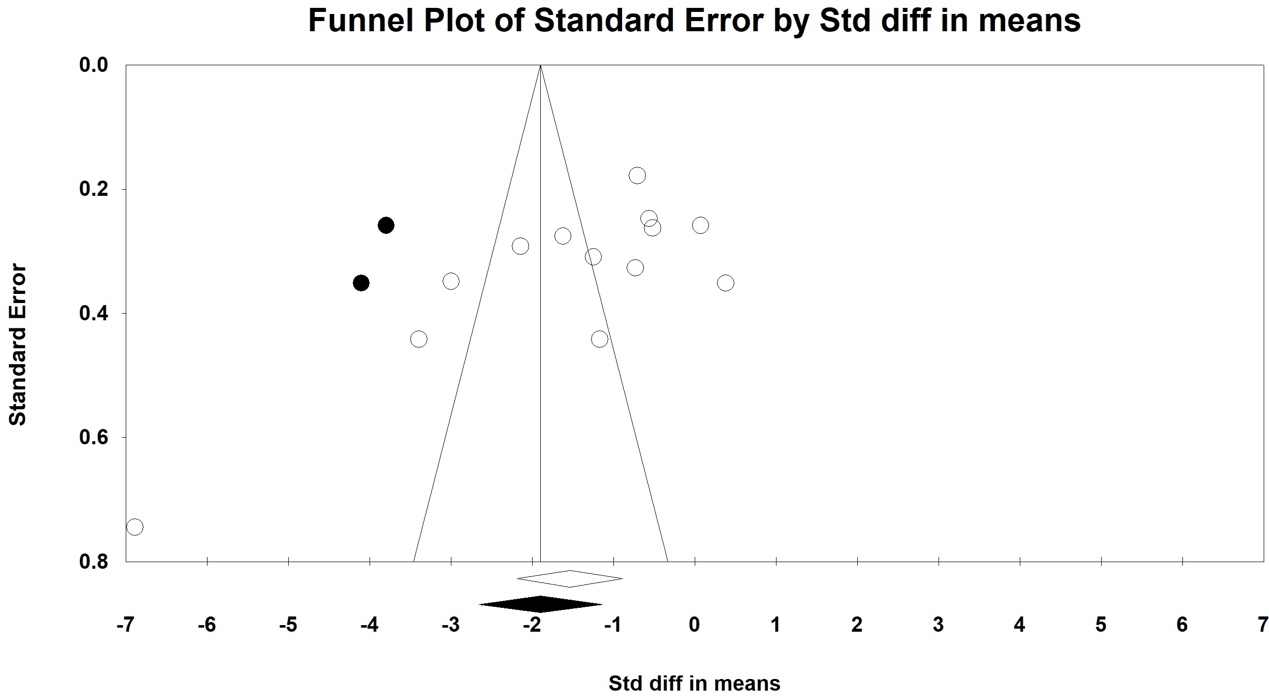


**Figure S3.** Duval and Tweedie’s Trim-and-Fill funnel plot for 0–24-hour postoperative analgesic requirements. The open circles represent the original observed studies, and the black circles denote two imputed (i.e., “missing”) studies to the left of the funnel’s mean effect. After imputing these additional points, the random-effects summary estimate shifted from an SMD of approximately −1.5 to −1.9, indicating that the true analgesic benefit of magnesium might be slightly stronger than initially observed. Nevertheless, the overall direction and statistical significance of the effect remained consistent. Std diff, standardized difference; SMD, standardized mean difference.

**Table S1.** Additional subgroup analyses for 0–24-hour postoperative analgesic requirements.

| Headline | Subgroup | Number of Trials^a^ | Mg group (*n*) | Control group (*n*) | Heterogeneity  (I^2^, %) | Effect estimate  SMD (95% CI)^b^ |
| --- | --- | --- | --- | --- | --- | --- |
| **0–24-hour postoperative analgesic requirements** | | | | | | |
| Geographic region | Asia | 9 | 302 | 300 | 91 | -1.24 (-1.85, -0.63) |
|  | Europe | 3 | 74 | 74 | 95 | -3.24 (-5.64, -0.84) |
|  | Americas | 1 | 16 | 17 | - | 0.37 (-0.32, 1.06) |
| Year published | 2001-2008 | 5 | 122 | 122 | 93 | -2.68 (-4.04, -1.32) |
|  | 2009-2016 | 2 | 50 | 50 | 0 | -0.59 (-1.00, -0.19) |
|  | 2017-2024 | 6 | 220 | 219 | 94 | -0.97 (-1.84, -0.11) |
| Surgery type | Body-cavity surgery | 4 | 110 | 110 | 91 | -1.50 (-2.55, -0.46) |
|  | Non-body-cavity surgery | 9 | 282 | 281 | 94 | -1.53 (-2.36, -0.70) |
| Mg administration | Bolus | 3 | 72 | 72 | 95 | -3.54 (-6.06, -1.03) |
|  | Continuous infusion | 1 | 30 | 30 | - | 0.07 (-0.43, 0.58) |
|  | Bolus followed by infusion | 9 | 290 | 289 | 89 | -1.13 (-1.68, -0.57) |
| Trial registration | Registered | 5 | 190 | 189 | 94 | -1.19 (-2.15, -0.22) |
|  | Not registered | 8 | 202 | 202 | 94 | -1.76 (-2.69, -0.82) |

^a^ Number of included trials that reported 0–24-hour analgesic requirements.

^b^ Random-effects model used for continuous data, reported as SMDs with 95% CIs.

CI: confidence interval; SMD: standardized mean difference.

**Table S2.** Meta-regression analyses for 0–24-hour postoperative analgesic requirements.

| Outcomes and moderators | Number of trials | Intercept | ß-coefficients | 95 % CI | *p*-value |
| --- | --- | --- | --- | --- | --- |
| **0–24-hour postoperative analgesic requirements** | | | | | |
| Age differences | 13 | -0.12 | -0.03 | -0.12~0.07 | 0.55 |
| Female ratio (gender differences) | 13 | -2.67 | 1.84 | -4.42~8.10 | 0.53 |
| Sample size | 13 | -1.79 | 0.01 | -0.04~0.05 | 0.78 |
| ASA classification | 12 | -0.17 | -0.52 | -2.97~1.92 | 0.65 |

Meta-regression analyses were conducted on 0–24‑hour postoperative analgesic requirements to assess the moderating effects of four measured factors: mean patient age, proportion of female participants, total sample size, and ASA classification. The results, summarized in this table, indicate no significant effect modification by any of the tested variables. CI, confidence interval; ASA, American Society of Anesthesiologists classification.
